# *Ganoderma lucidum* spore oil with *Ganoderma lucidum* and *Ganoderma sinense* extracts (G2SO) shows antitumor and Immunomodulatory effects in mice

**DOI:** 10.1038/s41598-025-34803-9

**Published:** 2026-01-05

**Authors:** Ricardo A. Wu-Chen, Chongxian Li, Changhui Wu, Kun Zhang, Liling Hong, Zucheng Ye, Cainan Wang, Jiancheng Xu, Xueyan Liu, Ye Li

**Affiliations:** 1 Fujian Xianzhilou Biological Science and Technology Co., Ltd, 6 Chuangxin Road, High-Tech Zone, Fuzhou, 350108 Fujian China; 2 School of Pharmacy, Fujian Medical University, Fuzhou, 350112 Fujian China; 3 Fujian Provincial Key Laboratory of Brain Aging and Neurodegenerative Diseases, The School of Basic Medical Sciences, Fujian Medical University, Fuzhou, 350112 Fujian China; 4 Institute of Food Science and Technology, College of Biological Science and Engineering, Fuzhou University, Fuzhou, 350108 Fujian China; 5 College of Chemical Engineering, Fuzhou University, Fuzhou, 350108 Fujian China

**Keywords:** *Ganoderma lucidum*, Fruiting body extracts, Antitumor activity, Immunomodulation, Medicine food homology, Cancer, Drug discovery, Immunology, Microbiology

## Abstract

*Ganoderma* is a highly valued medicinal fungus, especially in Traditional Chinese Medicine (TCM). A formulation of *Ganoderma lucidum* spore oil and aqueous extracts from the fruiting bodies of *Ganoderma lucidum* and *Ganoderma sinense* (G2SO) has been developed as a functional formulation proposed to support immune function and tumor control. The aim of this study was to evaluate the antitumor activity and immunomodulatory effects of G2SO in mice. A tumor-bearing mouse model was established using 4T1 cells in BALB/c mice for antitumor evaluation. Immunomodulatory outcomes were evaluated in ICR mice after 30 days of oral administration. The results showed that the inhibition rate of G2SO on 4T1 tumors in the tumor-bearing mice was 35.31 ± 9.51%. Moreover, G2SO produced small but statistically significant enhancements of innate immune function in mice, including natural killer (NK) cell cytotoxicity and macrophage phagocytosis. Indices of adaptive immunity (hemolysin production and ConA-induced T-cell proliferation) were also increased, whereas LPS-stimulated cytokines tumor necrosis factor-alpha (TNF-α), interleukin-6 (IL-6), and nitric oxide (NO) in RAW264.7 cells showed downward tendencies that were largely not significant. Overall, G2SO exhibits modest immunomodulatory activity together with measurable antitumor efficacy in vivo within the limits of the study design. These findings support further preclinical and clinical evaluation of G2SO as a functional formulation to support cancer prevention and immunomodulation.

**Supplementary Information:**

The online version contains supplementary material available at 10.1038/s41598-025-34803-9.

## Introduction

Research on health food has increased significantly in recent years over the concern of improving health through diet and nutrition. With rising awareness of the link between diet and chronic diseases, more consumers are turning to functional foods, nutraceuticals, and dietary supplements as part of a proactive approach to health management^1^.

*Ganoderma* is a highly valued fungus considered the “Mushroom of Immortality” in Traditional Chinese Medicine (TCM) due to its capacity to promote longevity and health^2^. *Ganoderma* is well-known to possess multiple bioactive effects, attributed to its content of bioactive compounds from the fruiting body and spores, including polysaccharides, triterpenes, steroids, ergosterols, nucleosides, alkaloids, amino acids, furan derivatives, and lipids^3–5^. Among the approximately 80 species of *Ganoderma* recorded in China, only *Ganoderma lucidum* and *Ganoderma sinense* are described as medicinally beneficial and has been used in TCM for more than 2,000 years^6^. These two *Ganoderma* species are classified under the TCM concept of “Medicine and Food Homology”, which posits that certain foods have medicinal properties and that there is no clear distinction between food and medicine^7^.

Previous studies have demonstrated that oral administration of *G. lucidum* spore oil can enhance immune activity in mice and prevent breast cancer metastasis^8^. These findings underscore the therapeutic potential of *Ganoderma*-based products and have paved the way for more detailed investigations into their mechanisms of action^9^. Building on this foundation, the development of a formulation of *G. lucidum* spore oil and aqueous extracts from the fruiting bodies of *G. lucidum* and *G. sinense* (G2SO) represents a significant advancement, offering improved bioavailability and potency. This product combines multiple active compounds of *Ganoderma* to enhance the immunomodulatory and anticancer effects^3,9^.

Breast cancer continues to be a leading cause of illness and death among women worldwide, representing a significant health challenge in recent years. Among its subtypes, triple-negative breast cancer (TNBC) is the most aggressive, lacking estrogen, progesterone, and HER2 receptors, which limits targeted treatment options and contributes to its poor prognosis. TNBC frequently metastasizes to bone, liver, brain, lungs, and other organs in advanced stages^10^. Breast cancer and the immune system interact in a highly intricate manner. Although immune defenses are designed to detect and eliminate tumor cells, breast cancer cells can develop ways to evade or suppress the immune response, allowing them to grow and spread^11^.

The immune system is integral to tumor surveillance, and enhancing immune responses, such as increasing cytokine production and activating natural killer (NK) cells, has been shown to improve outcomes in cancer models^12^. At the same time, direct anti-tumor effects, such as the induction of apoptosis and inhibition of tumor proliferation, remain crucial for effective cancer treatment^13^. However, there is a lack of integrated studies that examine both the immunomodulatory and therapeutic effects of a single agent in a comprehensive manner.

The present work investigates G2SO. This integrated formulation has not been previously evaluated in vivo. In this study, we aim to bridge that gap by investigating the dual role of G2SO in a murine model of breast cancer. Specifically, we examine how G2SO modulates immune function, potentially enhancing the body’s natural defenses, while also directly suppressing tumor growth. Additionally, we evaluate the acute toxicity of G2SO to confirm its safety profile, ensuring that its therapeutic benefits are not offset by adverse effects. By integrating these dimensions, immunopharmacology, antitumor efficacy, and toxicological evaluation, our study provides a more comprehensive understanding of G2SO’s therapeutic potential.

## Materials and methods

### Material

G2SO was provided by Fujian Xianzhilou Biological Science and Technology Co., Ltd. (Fuzhou, China). It is a softgel formulation (0.5 g per capsule) composed of a mixture of *G. lucidum* spore oil (extracted via supercritical CO₂ extraction)^8^ and aqueous extracts derived from the fruiting bodies of *G. lucidum* and *G. sinense*. The product specifications are as follows: each 100 g of G2SO contains 5 g crude polysaccharides, 21 g total triterpenes, and 20 mg of ganoderic acid A. The product is commercialized in dark capsules, each containing 0.5 g of G2SO, with a recommended dose of two capsules twice daily.

The supercritical CO_2_ extraction has been described in a previous study^8^. Dried, sporoderm-broken *G. lucidum* spore powder was subjected to supercritical CO₂ extraction to extract the oil. Extraction was carried out at a constant CO₂ flow rate of 20 L/h, under a system pressure of 4 MPa and a temperature of 36 °C, for a total duration of 2 h.

### Reagents

Mouse breast cancer cells 4T1 and YAC-1 cells were purchased from Shanghai Fuheng Biotechnology Co., Ltd. (Shanghai, China). RAW264.7 cells were purchased from Saiye Biotechnology Co., Ltd. (Guangzhou, China). Roswell Park Memorial Institute (RPMI) 1640 medium, Hank’s solution, Dulbecco’s Modified Eagle’s Medium (DMEM), trypsin-EDTA, and penicillin/streptomycin solution were purchased from Gibco (Waltham, MA). Fetal bovine serum (FBS) was purchased from PAN-Seratech (Aidenbach, Germany). Sodium carboxymethyl cellulose (CMC-Na) was purchased from Shanghai Changguang Enterprise Development Co., Ltd. (Shanghai, China). Palbociclib was purchased from MedChemExpress (MCE) (Monmouth Junction, NJ). Indian ink and Giemsa were purchased from Solarbio Technology Co., Ltd. (Beijing, China). Dinitrofluorobenzene (DNFB), sheep red blood cell (SRBC) suspension, and chicken red blood cells (CRBC) were purchased from Senbeijia (Nanjing, China). Thiazolyl blue tetrazolium bromide (MTT) solution, lipopolysaccharide (LPS), Concanavalin A (ConA), and sodium carbonate (Na_2_CO_3_) were purchased from Sigma-Aldrich (St. Louis, MO). Levamisole hydrochloride (LH) and cyclophosphamide (CTX) were purchased from Shanghai Yuanye Biotechnology Co., Ltd. (Shanghai, China). Dimethyl sulfoxide (DMSO) was purchased from Shanghai Macklin Biochemical Technology Co., Ltd. (Shanghai, China). Serum-free cell freezing medium CELLSAVING™ containing 10% DMSO was purchased from New Cell & Molecular Biotech Co., Ltd. (Suzhou, China). ELISA kits of interleukin-4 (IL-4) (RX203051M), interferon-γ (IFN-γ) (RX203097M), immunoglobulin A (IgA) (RX202739M), immunoglobulin M (IgM) (RX203431M), and immunoglobulin G (IgG) (RX202736M) were purchased from Ruixin Biotech (Quanzhou, China). ELISA kits of interleukin-6 (IL-6) (EK206/3) and tumor necrosis factor-α (TNF-α) (EK282/4) were purchased from Multi Sciences (Hangzhou, China). Nitric oxide (NO) assay kit was purchased from Beyotime Biotech (Shanghai, China).

### Determination of crude polysaccharides

Crude polysaccharides were determined using the Anthrone method^14^. Glucan was used as the reference to prepare the standard curve used to calculate the content of crude polysaccharides in the G2SO measured using an ultraviolet-visible (UV-Vis) spectrophotometer (Shimadzu UV-1800, Kyoto, Japan) at a wavelength of 625 nm.

### Determination of total triterpenes

Total triterpenes in G2SO were determined using the vanillin-perchloric acid method according to Lian et al.^8^. Ursolic acid was used as the reference to calculate the content of total triterpenes in G2SO measured using a UV-Vis spectrophotometer (Shimadzu UV-1800, Kyoto, Japan) at a wavelength of 548 nm.

### Determination of Ganoderic acid A

An amount of 1 g G2SO sample was diluted in 50 mL methanol and sonicated. The determination of ganoderic acid A was carried out using high-performance liquid chromatography (HPLC) (Waters Alliance e2695, Waters Corp., Milford, MA) equipped with a 2998 photodiode array (PDA) detector. Chromatographic separation was performed in an Agilent Zorbax Plus C18 column (150 mm × 4.6 mm, 5 μm) kept at 35 °C. The mobile phase consisted of acetonitrile (A), methanol (B), and 0.4% formic acid aqueous solution (C) at a flow rate of 1.0 mL/min. The gradient elution was: 0–30 min, 20% A, 20% B, 60% C; 30–35 min, 30% A, 20% B, 50% C; 35–40 min, 38% A, 20% B, 42% C; 40–45 min, 20% A, 20% B, 60% C. The injection volume was 20 µL and the PDA absorbance wavelength was set at 254 nm.

### Determination of ergosterol

The determination of ergosterol content was carried out according to Lian et al.^8^ with some modifications, using an HPLC (Shimadzu Prominence, Shimadzu Corp., Kyoto, Japan) equipped with a UV-Vis detector. G2SO sample (0.1 g) was diluted in 25 mL chloroform/methanol solution (1:1 v/v). Separation was performed in an ODS-2 Hypersil C18 column (250 mm × 4.6 mm, 5 μm) kept at 35 °C. The mobile phase consisted of 100% methanol at a flow rate of 1.0 mL/min from 0 to 15 min, 1.6 mL/min from 18 to 55 min, and 1.0 mL/min from 56 to 65 min. The injection volume was 10 µL and the UV-Vis absorbance wavelength was set at 282 nm.

### Batch-to-batch consistency of G2SO

Batch-to-batch consistency of G2SO was evaluated using the four chemical markers described above, namely crude polysaccharides, total triterpenes, ganoderic acid A and ergosterol. A total of 32 industrial batches of G2SO were analysed. For each marker, the minimum, maximum, range, mean, standard deviation (SD) and coefficient of variation (CV, %) across the 32 batches were calculated using GraphPad Prism 9 (GraphPad Software, Boston, MA), where CV (%) = (SD/mean) × 100. These parameters were used to describe the inter-batch variability of the G2SO formulation.

### In vivo antitumor evaluation

#### Cell culture

Mouse breast cancer cells 4T1 were cultured in RPMI 1640 medium supplemented with 10% FBS and 1% (v/v) penicillin/streptomycin. Cells were incubated at 37 °C in a humidified atmosphere containing 5% CO₂. Cultures were monitored daily and passaged every 2–3 days when they reached ~ 80% confluency, using 0.25% trypsin-EDTA for detachment. For cryopreservation, cells were resuspended in serum-free CELLSAVING™ cell freezing medium and stored in -80 °C.

#### Animals and experimental design

Six-week-old female BALB/c (16–18 g) were obtained from Guangdong Yaokang Biotechnology Co., Ltd. (Foshan, China). To establish the 4T1 tumor model, logarithmic-phase 4T1 cells were collected and washed twice with PBS and resuspended at 1 × 10^7^ cells/mL^15^. Afterwards, 0.2 mL of this suspension was subcutaneously injected into the right axillary region of each mouse. Six days after implantation, tumors reached 50–100 mm^3^. To minimize bias, mice were randomized by baseline tumor volume immediately before dosing into three experimental groups: (1) control (*n* = 10), receiving 0.5% CMC-Na (vehicle); (2) positive control (*n* = 10), receiving palbociclib at 100 mg/kg bw/d; and (3) G2SO group (*n* = 10), receiving G2SO at 1 g/kg bw/d. All treatments were administered by oral gavage in a 0.5% CMC-Na suspension for 21 days. Humane endpoints limited tumor growth to ≤ 2.0 cm in any dimension or to an estimated total tumor volume equivalent to ≤ 10% of body weight, whichever occurred first. Tumor volume (mm^3^) was calculated using the formula described in 2.7.3. To express tumor burden as a percentage of body weight (g) measured on the same day, we used the standard approximation of tumor density ≈ 1 g/cm³ (i.e., 1,000 mm³ ≈ 1 g): %BW=((volume/1000) ÷ body weight)×100 Animals were monitored twice daily. Health status was assessed by body weight (every other day), food and water intake, and cage-side observations of activity, respiration, and coat condition. At the study endpoint, mice were deeply anesthetized with sodium pentobarbital (2% w/v, 100 mg/kg intraperitoneal injection), followed by terminal blood collection (orbital sinus puncture) and euthanized by cervical dislocation.

#### Measurement of tumor volume and tumor Inhibition rate

Tumor volume was measured with digital calipers every two days using the following formula:

$$\:\mathrm{T}\mathrm{u}\mathrm{m}\mathrm{o}\mathrm{r}\:\mathrm{v}\mathrm{o}\mathrm{l}\mathrm{u}\mathrm{m}\mathrm{e}\:\left({\mathrm{m}\mathrm{m}}^{3}\right)=({\mathrm{a}}^{2}\times\:\mathrm{b})/2$$

Where: a is the tumor’s short diameter and b is the tumor’s long diameter in mm.

On day 21, tumor-bearing mice were sacrificed, and the tumors were dissected and weighed. The tumor inhibition rate was calculated using the following formula:

$$\:\mathrm{I}\mathrm{R}\left(\mathrm{\%}\right)=\left[\right({\mathrm{W}}_{\mathrm{c}\mathrm{o}\mathrm{n}\mathrm{t}\mathrm{r}\mathrm{o}\mathrm{l}}-{\mathrm{W}}_{\mathrm{d}\mathrm{r}\mathrm{u}\mathrm{g}})/{\mathrm{W}}_{\mathrm{c}\mathrm{o}\mathrm{n}\mathrm{t}\mathrm{r}\mathrm{o}\mathrm{l}}]\times\:100$$

Where: IR is the tumor inhibition rate, W_drug_ is the tumor weight in the treatment group and W_control_ is the tumor weight in the control group in g.

#### Measurement of body weight

Beginning on the first day of intragastric administration, the tumor-bearing mice were weighed at a consistent time, and these measurements were used to construct a body weight change curve.

#### Blood cell counts and serum biochemical analysis

Approximately 1.0–1.4 mL of blood was collected per mouse. Half of the sample was transferred into tubes containing anticoagulant (EDTA) and centrifuged at 10 000 × g for 10 min for blood cell counts. The remaining blood was placed in plain Eppendorf tubes, allowed to clot at room temperature for 30 min, and then centrifuged at 3 000 × g for 10 min to obtain serum for biochemical analysis. Biochemical analysis included aspartate aminotransferase (AST), alanine aminotransferase (ALT), urea (URE), and creatinine (CRE).

### In vivo evaluation of immune-enhancing effects in normal mice

#### Animals and experimental design

Six-week-old male ICR mice (18–22 g) were obtained from Guangdong Yaokang Biotechnology Co., Ltd. (Foshan, Guangdong, China). The number of animals used and experimental procedures were carried out in accordance with Technical Standards for Testing and Assessment of Health Food in China^16^. We employed male ICR mice to avoid the known cyclic variations in female sex hormones that can influence innate and adaptive immune readouts^17^.

Mice were assigned to five experimental groups based on body weight. Mice received 0.167, 0.333, and 1.0 g/kg body weight of G2SO designated as low (G2SO-L), medium (G2SO-M), and high (G2SO-H) doses, respectively. G2SO was dissolved in vegetable oil and administered via gavage once daily for 30 days. Animals were monitored twice daily. Health status was assessed by body weight (every other day), food and water intake, and cage-side observations of activity, respiration, and coat condition. At the study endpoint, mice were deeply anesthetized with sodium pentobarbital (2% w/v, 100 mg/kg intraperitoneal injection), followed by terminal blood collection (orbital sinus puncture) and euthanized by cervical dislocation.

The five Groups were designed for different immunological assays:

Group I – plaque assay, serum hemolysin, organ index.

Group II – splenic lymphocyte proliferation and NK cell activity.

Group III – delayed-type hypersensitivity.

Group IV – macrophage phagocytosis (carbon clearance).

Group V – peritoneal macrophage phagocytosis.

Each Group included four subgroups receiving G2SO-L (*n* = 10), G2SO-M (*n* = 10), G2SO-H (*n* = 10), or vehicle control (*n* = 10).

When interpreting the results, body surface area allometric scaling should be considered when converting to the human equivalent dose (HED) with K_m_ values of 3 for mouse and 37 for adult human^18^. Thus, the effect of the study doses of 0.167, 0.333, and 1.0 g/kg in mice correspond to 13.5, 26.9, and 81 mg/kg HED, respectively. For a 60-kg adult these approximate to 0.81, 1.61, and 4.86 g/day. These values are provided to contextualize exposure only and are not recommended human doses.

#### Macrophage phagocytosis by carbon clearance method

The macrophage phagocytic index was measured using the carbon clearance method according to Zhu et al.^19^ with some modifications. Briefly, mice were injected with 10 mL/kg bw of Indian ink suspension through the tail vein. Blood from the retro-orbital plexuses of mice was collected after 2 and 10 min after injection and then added to 2 mL of 0.1% Na_2_CO_3_ solution. The optical density (OD) of the suspension was measured using a UV-Vis spectrophotometer (Shimadzu UV-1800, Kyoto, Japan) at a wavelength of 600 nm. Na_2_CO_3_ solution was used as the control. The phagocytic index was calculated using the following equation:

$$\:\mathrm{P}\mathrm{h}\mathrm{a}\mathrm{g}\mathrm{o}\mathrm{c}\mathrm{y}\mathrm{t}\mathrm{i}\mathrm{c}\:\mathrm{i}\mathrm{n}\mathrm{d}\mathrm{e}\mathrm{x}=\frac{\mathrm{B}\mathrm{W}}{\mathrm{l}\mathrm{i}\mathrm{v}\mathrm{e}\mathrm{r}\:\mathrm{w}\mathrm{e}\mathrm{i}\mathrm{g}\mathrm{h}\mathrm{t}\:+\:\mathrm{s}\mathrm{p}\mathrm{l}\mathrm{e}\mathrm{e}\mathrm{n}\:\mathrm{w}\mathrm{e}\mathrm{i}\mathrm{g}\mathrm{h}\mathrm{t}}\times\:\sqrt[3]{\frac{{\mathrm{l}\mathrm{g}\mathrm{O}\mathrm{D}}_{1}-{\mathrm{l}\mathrm{g}\mathrm{O}\mathrm{D}}_{2}}{{\mathrm{t}}_{2}-{\mathrm{t}}_{1}}}$$

Where OD_1_ and OD_2_ are the ODs at times t_1_ (2 min) and t_2_ (10 min), respectively.

#### Delayed type hypersensitivity (DTH) response

The DTH test was performed with modifications based on Zhu et al.^19^. Briefly, a 3 × 3 cm area on each mouse’s abdomen was shaved, and 50 µL of 1% DNFB in acetone–seed oil (1:1) was applied for sensitization. Five days later, 10 µL of 1% DNFB was applied to the right ear, while the left ear remained untreated. After 24 h, mice were euthanized by cervical dislocation, and 8 mm ear punches were collected and weighed. Ear swelling was calculated as the weight difference between the right and left ears.

#### Hemolysin-producing cell assay and levels of serum hemolysin assay

Mice were intraperitoneally injected with 2% (v/v) SRBC suspension (1 × 10⁹ cells/mL). After 5 days, blood was collected for the serum hemolysin assay, and spleens were harvested for the hemolysin-producing cell assay.

For the latter, spleens were ground in Hank’s solution, filtered, centrifuged, and resuspended at 5 × 10⁶ cells/mL. Surface medium was prepared by mixing agarose in double-distilled water with 2× Hank’s solution and heating to 45–50 °C. Then, 0.5 mL of medium, 50 µL of 10% SRBC, and 25 µL of spleen cell suspension were combined and spread on glass slides. After solidification and 1.5 h incubation, guinea pig serum was added, followed by a second incubation. Hemolytic plaques were then counted.

For the serum hemolysin assay, blood was coagulated for 1 h, and serum was collected by centrifugation. Serial 2-fold dilutions of serum (100 µL) were mixed with 100 µL of 0.5% SRBC in a microplate. After 3 h at 37 °C, hemagglutination was observed, and antibody levels were calculated using the following equation:

$$\:\mathrm{A}\mathrm{n}\mathrm{t}\mathrm{i}\mathrm{b}\mathrm{o}\mathrm{d}\mathrm{y}\:\mathrm{l}\mathrm{e}\mathrm{v}\mathrm{e}\mathrm{l}={\mathrm{S}}_{1}+{2\mathrm{S}}_{2}+{3\mathrm{S}}_{3}+...+{\mathrm{n}\mathrm{S}}_{\mathrm{n}}$$

Where 1,2,3…, n, represent the index of double dilution, S represents the level of agglutination.

#### Phagocytosis of peritoneal macrophages

Phagocytosis by peritoneal macrophages was evaluated with modifications based on Huang et al.^20^. Mice were intraperitoneally injected with 1 mL of 20% CRBC and sacrificed 30 min later. Then, 2 mL of saline was injected, and the abdomen was gently pressed for 20 s. Peritoneal lavage fluid (1 mL) was collected, spread on two glass slides, and incubated at 37 °C for 30 min. After washing with saline, slides were air-dried, fixed in acetone–methanol (1:1), and stained with 4% Giemsa for 3 min. After rinsing and drying, macrophages were observed under oil immersion, and the phagocytic percentage and index were calculated using the following formulas:

$$\:\mathrm{P}\mathrm{e}\mathrm{r}\mathrm{c}\mathrm{e}\mathrm{n}\mathrm{t}\mathrm{a}\mathrm{g}\mathrm{e}\:\mathrm{p}\mathrm{h}\mathrm{a}\mathrm{g}\mathrm{o}\mathrm{c}\mathrm{y}\mathrm{t}\mathrm{o}\mathrm{s}\mathrm{i}\mathrm{s}=\frac{\mathrm{n}\mathrm{u}\mathrm{m}\mathrm{b}\mathrm{e}\mathrm{r}\:\mathrm{o}\mathrm{f}\:\mathrm{p}\mathrm{h}\mathrm{a}\mathrm{g}\mathrm{o}\mathrm{c}\mathrm{y}\mathrm{t}\mathrm{i}\mathrm{c}\:\mathrm{c}\mathrm{e}\mathrm{l}\mathrm{l}\mathrm{s}\:\mathrm{i}\mathrm{n}\mathrm{g}\mathrm{e}\mathrm{s}\mathrm{t}\mathrm{i}\mathrm{n}\mathrm{g}\:\mathrm{C}\mathrm{R}\mathrm{B}\mathrm{C}}{\mathrm{n}\mathrm{u}\mathrm{m}\mathrm{b}\mathrm{e}\mathrm{r}\:\mathrm{o}\mathrm{f}\:\mathrm{m}\mathrm{a}\mathrm{c}\mathrm{r}\mathrm{o}\mathrm{p}\mathrm{h}\mathrm{a}\mathrm{g}\mathrm{e}\mathrm{s}}\times\:100$$

$$\:\mathrm{P}\mathrm{h}\mathrm{a}\mathrm{g}\mathrm{o}\mathrm{c}\mathrm{y}\mathrm{t}\mathrm{i}\mathrm{c}\:\mathrm{i}\mathrm{n}\mathrm{d}\mathrm{e}\mathrm{x}=\frac{\mathrm{n}\mathrm{u}\mathrm{m}\mathrm{b}\mathrm{e}\mathrm{r}\:\mathrm{o}\mathrm{f}\:\mathrm{p}\mathrm{h}\mathrm{a}\mathrm{g}\mathrm{o}\mathrm{c}\mathrm{y}\mathrm{t}\mathrm{i}\mathrm{z}\mathrm{e}\mathrm{d}\:\mathrm{C}\mathrm{R}\mathrm{B}\mathrm{C}}{\mathrm{n}\mathrm{u}\mathrm{m}\mathrm{b}\mathrm{e}\mathrm{r}\:\mathrm{o}\mathrm{f}\:\mathrm{m}\mathrm{a}\mathrm{c}\mathrm{r}\mathrm{o}\mathrm{p}\mathrm{h}\mathrm{a}\mathrm{g}\mathrm{e}\mathrm{s}}$$

#### Proliferation and NK cell cytotoxicity of splenocytes

Mice were sacrificed, and spleens were ground through a 200 μm cell strainer. The cell suspension was washed, centrifuged, and resuspended in RPMI 1640 medium to obtain splenocytes. The suspension was divided for proliferation and NK cytotoxicity assays.

The thiazolyl blue tetrazolium bromide (MTT) assay was used to assess splenocyte proliferation. Splenocyte suspensions (1 mL) were added to two wells of a 24-well plate. ConA (75 µL, 7.5 µg/mL) was added to one well, and the other served as the control. Cells were cultured for 72 h at 37 °C. Four hours before the end of incubation, MTT solution (50 µL, 5 mg/mL) was added. After 4 h, acidic propanol (1 mL) was added to dissolve the formazan crystals, and OD was measured at 570 nm^21^.

NK activity was measured using an lactate dehydrogenase (LDH) release assay^16^. Splenocytes (5 × 10^6^ cells/mL) and YAC-1 cells (1 × 10^5^ cells/mL) were mixed (50:1 ratio) in a 96-well plate. Controls included spontaneous and maximum release conditions. After 4 h of incubation at 37 °C, supernatants were transferred to a new plate with LDH substrate, incubated for 5 min, and the reaction stopped with 30 µL of 1 M hydrochloric acid (HCl). Absorbance was measured at 490 nm^21^.

$$\:\mathrm{N}\mathrm{K}\:\mathrm{c}\mathrm{e}\mathrm{l}\mathrm{l}\:\mathrm{a}\mathrm{c}\mathrm{t}\mathrm{i}\mathrm{v}\mathrm{i}\mathrm{t}\mathrm{y}\:\left(\mathrm{\%}\right)=\frac{{\mathrm{O}\mathrm{D}}_{\mathrm{s}\mathrm{a}\mathrm{m}\mathrm{p}\mathrm{l}\mathrm{e}}-{\mathrm{O}\mathrm{D}}_{\mathrm{Y}\mathrm{A}\mathrm{C}-1\:\mathrm{c}\mathrm{e}\mathrm{l}\mathrm{l}\:\mathrm{s}\mathrm{p}\mathrm{o}\mathrm{n}\mathrm{t}\mathrm{a}\mathrm{n}\mathrm{e}\mathrm{o}\mathrm{u}\mathrm{s}\:\mathrm{r}\mathrm{e}\mathrm{l}\mathrm{e}\mathrm{a}\mathrm{s}\mathrm{e}}}{{\mathrm{O}\mathrm{D}}_{\mathrm{Y}\mathrm{A}\mathrm{C}-1\:\mathrm{c}\mathrm{e}\mathrm{l}\mathrm{l}\:\mathrm{m}\mathrm{a}\mathrm{x}\mathrm{i}\mathrm{m}\mathrm{u}\mathrm{m}\:\mathrm{r}\mathrm{e}\mathrm{l}\mathrm{e}\mathrm{a}\mathrm{s}\mathrm{e}}}\times\:100$$

#### Measurement of immune organ indices

Twenty four hours after the last administration of G2SO, the mice were weighed and sacrificed. The spleen and thymus indices were calculated using the following formula:

$$\:\mathrm{i}\mathrm{n}\mathrm{d}\mathrm{e}\mathrm{x}\:\left(\mathrm{\%}\right)=(\mathrm{w}\mathrm{e}\mathrm{i}\mathrm{g}\mathrm{h}\mathrm{t}\:\mathrm{o}\mathrm{f}\:\mathrm{t}\mathrm{h}\mathrm{y}\mathrm{m}\mathrm{u}\mathrm{s}\:\mathrm{o}\mathrm{r}\:\mathrm{s}\mathrm{p}\mathrm{l}\mathrm{e}\mathrm{e}\mathrm{n}/\mathrm{b}\mathrm{o}\mathrm{d}\mathrm{y}\:\mathrm{w}\mathrm{e}\mathrm{i}\mathrm{g}\mathrm{h}\mathrm{t})\times\:100$$

### Exploratory in vivo evaluation of immune-enhancing effects in immunosuppressed mice

CTX was used to induce transient immunosuppression as a surrogate for chemotherapy-associated myelosuppression. CTX-treated mice were included to test whether G2SO restores immune function under cytotoxic stress and to assess safety in immunocompromised conditions.

#### Animals and experimental design

Six-week-old male ICR mice (18–22 g) were obtained from Guangdong Yaokang Biotechnology Co., Ltd. (Foshan, Guangdong, China). Mice were randomly divided into six groups after 7 days acclimatization period as follows: vehicle control group (untreated, vegetable oil), model group (CTX) (*n* = 10), G2SO-L (*n* = 10), G2SO-M (*n* = 10), G2SO-H (*n* = 10), and LH group (positive control, 10 mg/kg bw) (*n* = 10). The positive control (LH) group was included solely in this exploratory experiment to validate the CTX model and benchmark G2SO’s protective effects.

The mice were orally administered with the above treatments for 30 days. On the 27th day, the mice in all groups except the control group were intraperitoneally injected with 80 mg/kg CTX for 3 days. The body weights of the mice were monitored every two days. On day 30th, mice were sacrificed and organ indices of the spleen and thymus were calculated. Mice were anesthetized by intraperitoneal injection of sodium pentobarbital at 100 mg/kg bw prior to blood withdrawal and sacrifice. Animals were monitored twice daily. Health status was assessed by body weight (every other day), food and water intake, and cage-side observations of activity, respiration, and coat condition. At the study endpoint, mice were deeply anesthetized with sodium pentobarbital (2% w/v, 100 mg/kg intraperitoneal injection), followed by terminal blood collection (orbital sinus puncture) and euthanized by cervical dislocation.

#### Levels of serum cytokines by ELISA

Blood serum was collected as described in 2.7.5. Levels of IL-4, IFN-γ, IgA, IgM, and IgG in blood serum were detected by ELISA assay kits following the manufacturer’s instructions.

#### Histopathological examination

Freshly collected spleen and thymus were fixed in 4% paraformaldehyde (PFA) solution for 24 h. The tissues were trimmed and dehydrated, then embedded in paraffin. Once the paraffin had solidified, the blocks were trimmed, frozen overnight at − 20 °C, and sectioned. The sections were transferred onto slides, stained with hematoxylin and eosin (H&E), and observed under an optical microscope.

Spleen and thymus sections were assessed for vacuolization, cellular swelling, vascular congestion and inflammatory cell infiltration. Lesions were graded using a semi-quantitative scoring system adapted from Sun et al.^22^: 0, no detectable damage; 1, < 10% of the field showing the above lesions; 2, 10–30% of the field affected; 3, 30–60% of the field affected; and 4, > 60% of the field affected. The mean score of all fields examined was taken as the final histological score for each mouse.

### In vitro evaluation using RAW264.7 cells

#### Cell culture

RAW264.7 cells were cultured in DMEM supplemented with 10% FBS, 100 U/mL penicillin, and 100 U/mL streptomycin, incubated in a humidified incubator at 37 °C with 5% CO_2_ and monitored daily. Medium was refreshed every 2–3 days, and cells were passaged at approximately 70–80% confluency using 0.25% trypsin–EDTA at a split ratio of 1:3. Experiments were carried out when the cells were in logarithmic phase. For cryopreservation, cells were resuspended in serum-free CELLSAVING™ cell freezing medium and stored at -80 °C.

#### Cell viability

RAW264.7 cells were seeded at 1 × 10⁴/well in 96-well plates and allowed to adhere for 24 h at 37 °C in a 5% CO_2_ incubator. Cells were then treated with G2SO (62.5, 125, 250, 500, 1000, 2000 µg/mL), LH (10 nmol/mL), and LPS (1 µg/mL) for another 24 h. Cell viability was assessed by the MTT method according to our previous report with some modifications^23^. Briefly, 20 µL of 5 mg/mL MTT solution (in PBS) was added to each well and plates were incubated for 4 h. Following removal of the supernatant, the formazan crystals were dissolved in 150 µL DMSO with gentle agitation for 15 min. Absorbance was measured at 540 nm using a microplate reader. Cell viability (%) was expressed as a percentage of control (set to 100%).

#### Determination of the adhesion function of RAW264.7 cells

The effect of G2SO on RAW264.7 cell adhesion was assessed following Li et al.^24^. RAW264.7 cells (1 × 10⁶/well) were seeded in 6-well plates and incubated for 24 h, then treated with G2SO at 125, 250, 500, or 1000 µg/mL. After treatment, cells were centrifuged, collected, and reseeded in 24-well plates (2.5 × 10⁵/well) for 1 h. Cells were then washed with PBS, fixed in 4% PFA (0.5 mL, 15 min), washed again, and stained with 0.5% crystal violet for 15 min. After a final PBS wash, cells were observed and imaged under an inverted microscope at 100× magnification.

#### Determination of cytokines of RAW264.7 cells

RAW264.7 cells at 80% confluence were harvested, adjusted to 1 × 10⁶ cells/mL, and seeded in 96-well plates for 24 h. Cells were then treated with G2SO (125–1000 µg/mL) and LPS (1 µg/mL) for another 24 h. Supernatants were collected, and IL-6, TNF-α, and NO levels were measured using ELISA and Griess assay kits following the manufacturers’ instructions.

### Statistical analysis

The statistical analysis was carried out using GraphPad Prism 9 (GraphPad Software, Boston, MA). Results are expressed as mean ± SD. For in vivo experiments, *n* denotes the number of animals per group (biological replicates). For in vitro assays, *n* denotes the number of independent experiments, each performed with three technical replicates (wells); the mean of the technical replicates was used as a single value for statistical analysis. Group differences were evaluated by one-way ANOVA; Dunnett’s test was used for multiple comparisons. Datasets were tested for normality using the Shapiro–Wilk test to confirm normal distribution prior to ANOVA. Homogeneity of variances was evaluated with the Brown-Forsythe test at α = 0.05 for each endpoint; Bartlett’s test was also tabulated as a sensitive check. A *P* value of ≤ 0.05 was considered statistically significant. For each dataset, *n* and the error bar type are specified in the corresponding legends.

## Results

### Characterization of G2SO

The main bioactive compounds in G2SO are polysaccharides, triterpenes, ganoderic acid A, and ergosterol. The compound that exhibited the highest concentration was total triterpenes with 33.73 ± 2.00 g/100 g, followed by crude polysaccharides (7.82 ± 0.37 g/100 g), ergosterol (223.4 ± 13.12 mg/100 g), and ganoderic acid A (84.63 ± 8.55 mg/100 g) (Table 1).

**Table 1:** Contents of main bioactive compounds in G2SO and batch-to-batch consistency information.

Parameter	Crude polysaccharides (g/100 g)	Total triterpenes (g/100 g)	Ganoderic acid A (mg/100 g)	Ergosterol (mg/100 g)
Number of batches*	32	32	32	32
Minimum	6.9 g/100 g	31 g/100 g	72 mg/100 g	199 mg/100 g
Maximum	8.6 g/100 g	39.80 g/100 g	101 mg/100 g	267 mg/100 g
Range	1.7	8.8	29	68
Mean	7.82 g/100 g	33.73 g/100 g	84.63 mg/100 g	223.4 mg/100 g
Standard deviation	0.37	2.00	8.55	13.12
Standard error of mean	0.07	0.35	1.51	2.32
Coefficient of variation	4.71%	5.94%	10.11%	5.87%

*Detailed batch information in Supplementary Table S1.

To evaluate the batch-to-batch consistency of G2SO, these four quality markers were quantified in 32 independent batches (Table 1; Supplementary Table S1). The contents showed narrow ranges (polysaccharides: 6.9–8.6 g/100 g; triterpenes: 31–39.8 g/100 g; ganoderic acid A: 72–101 mg/100 g; ergosterol: 199–267 mg/100 g) and low inter-batch coefficients of variation (4.71%, 5.94%, 10.11%, and 5.87%, respectively).

### In vivo antitumor evaluation

#### Changes in body weight of tumor-bearing mice

**Fig. 1:**
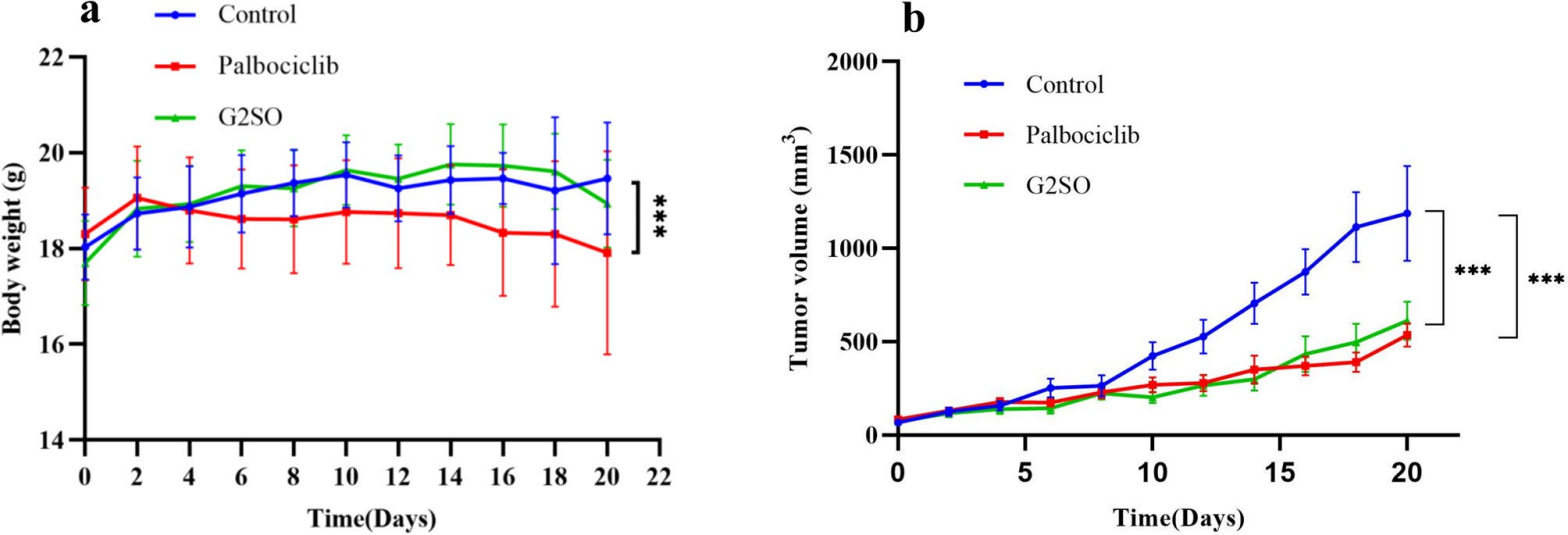
Changes in body weight (**a**) and tumor volume (**b**) of tumor-bearing mice during treatment. Data are expressed as mean ± SD. The dose of Palbociclib was 100 mg/kg bw/d and G2SO was 1 g/kg bw/d oral gavage. *n* = 10. These treatments were compared to the control. ***P < 0.001.

The effects of G2SO on the body weight of tumor-bearing mice are shown in Fig. 1a. The weight change observed in the positive control group (Palbociclib) (17.91 ± 2.12 g) was statistically significant (*P* < 0.001) compared to the control group (19.46 ± 1.17 g), whereas no significant differences were found between the groups treated with G2SO (18.94 ± 0.91 g) and the control group.

#### Effect of G2SO on tumor volume

To study the general effect of G2SO on tumor-bearing mice, the tumor volume was recorded after 21 days of treatment. Palbociclib (535.52 ± 187.91 mm^3^) and G2SO (611.69 ± 322.49 mm^3^) groups displayed significantly lower (*P* < 0.001) tumor volume compared to the control (1187.02 ± 720.05 mm^3^) (Fig. 1b), suggesting that G2SO treatment suppressed tumor growth at levels comparable to Palbociclib.

#### Effect of G2SO on tumor weight and inhibition-rate in tumor-bearing mice

**Fig. 2:**
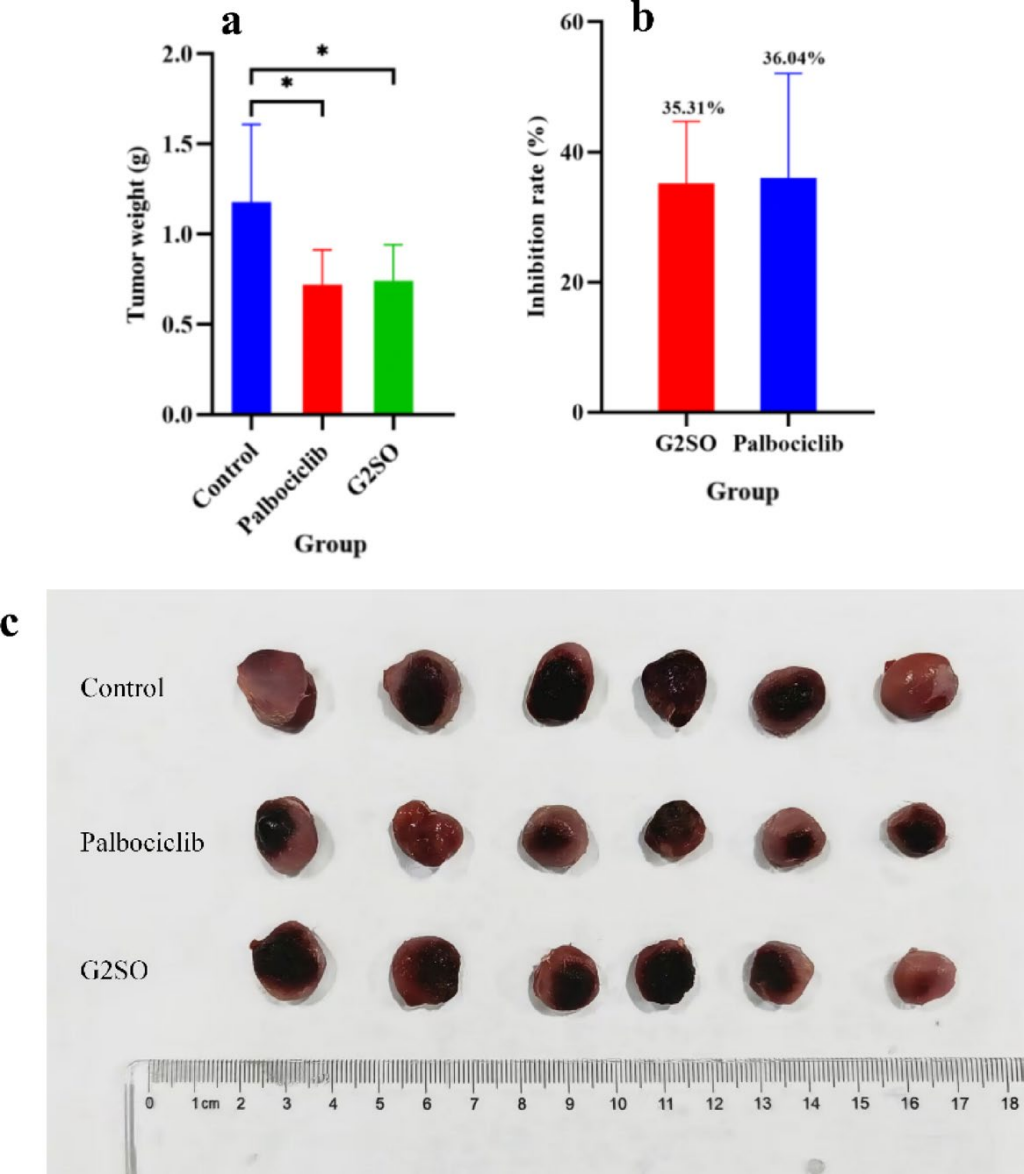
Effects of G2SO on tumor growth in 4T1 tumor-bearing mice. Tumor weight (**a**), inhibitory rate (**b**), and tumor size comparison (**c**) of 4T1 influenced by G2SO treatment. Largest tumor diameter at day 21 (endpoint) was ~ 1.4 cm. Data are expressed as mean ± SD. Treatment groups: Control: vehicle only; G2SO: 1 g/kg bw/d oral gavage; Palbociclib: 100 mg/kg bw/d oral gavage. *n* = 10. G2SO and Palbociclib groups were compared to the control. *P < 0.05.

After 21 days of treatment, tumors were dissected and weighed (Fig. 2c). Tumor weight of the control group, Palbociclib group, and G2SO group were 1.178 ± 0.430, 0.721 ± 0.192, and 0.679 ± 0.201 g, respectively. The tumor weights of Palbociclib and G2SO groups were significantly (*P* < 0.05) lower than that of the control group (Fig. 2a). The inhibition rates of Palbociclib group and G2SO group were 36.04 ± 16.06% and 35.31 ± 9.51%, respectively (Fig. 2b).

#### Effect of G2SO on blood cell counts and serum biochemical analysis

Blood count analysis revealed that white blood cells (WBC), lymphocytes (LYM), monocytes (MONO), and neutrophils (NEUT) counts of Palbociclib group (50.99 ± 29.53, 7.89 ± 4.28, 2.98 ± 2.10, 36.00 ± 27.89, respectively) were significantly (*P* < 0.05) lower than those of control group (148.61 ± 63.58, 32.99 ± 24.62, 8.03 ± 2.04, 107.6 ± 72.20, respectively) (Table 2). Moreover, except for NEUT count (33.27 ± 33.39), G2SO group did not show significant differences in the blood cell counts compared to the control.

**Table 2:** Blood cell counts of tumor-bearing mice (mean ± SD).

Parameter	Unit	Control	Palbociclib	G2SO
WBC	10^9^/L	148.61 ± 63.58	50.99 ± 29.53^*^	143.24 ± 99.89
LYM	10^9^/L	32.99 ± 24.62	7.89 ± 4.28^*^	31.49 ± 24.45
MONO	10^9^/L	8.03 ± 2.04	2.98 ± 2.10^**^	7.02 ± 4.42
NEUT	10^9^/L	107.6 ± 72.20	36.00 ± 27.89^*^	33.27 ± 33.39^*^
RBC	10^9^/L	6.67 ± 0.34	6.21 ± 0.57	6.53 ± 0.99
HGB	g/L	122.86 ± 1.35	121.78 ± 11.45	121.88 ± 16.51
PLT	10^9^/L	752.29 ± 99.18	640.89 ± 165.27	566.25 ± 251.11

The dose of Palbociclib was 100 mg/kg bw/d and G2SO was 1 g/kg bw/d. Compared to the control: *P < 0.05;**P < 0.01.

WBC: white blood cells; LYM: lymphocytes; MONO: monocytes; NEUT: neutrophils; RBC: red blood cells; HGB: hemoglobin; PLT: platelets.

Control = normal (non-tumor) mice.

The levels of biochemical parameters related to liver function (ALT and AST) and renal function (URE and CRE) did not show significant (*P* > 0.05) differences after G2SO administration compared to the control group (normal non-tumor mice) (Table 3).

**Table 3:** Parameters of serum biochemical analysis of tumor-bearing mice (mean ± SD).

Parameter	Unit	Control	Palbociclib	G2SO
ALT	U/L	36.06 ± 8.73	25.58 ± 6.70*	43.19 ± 8.92
AST	U/L	202.20 ± 48.03	175.06 ± 27.96	255.06 ± 103.34
URE	µmol/L	17.90 ± 7.42	14.28 ± 2.53	22.41 ± 6.29
CRE	mg/dL	16.20 ± 2.39	16.46 ± 3.29	19.01 ± 3.34

The dose of Palbociclib was 100 mg/kg bw/d and G2SO was 1 g/kg bw/d. Compared to the control: *P < 0.05.

ALT: Alanine aminotransferase; AST: Aspartate aminotransferase; URE: urea; CRE: creatinine.

Control = normal (non-tumor) mice.

### In vivo evaluation of immune-enhancing effects in normal mice

#### Effect of G2SO on body weight and immune organs indices in normal mice

**Fig. 3:**
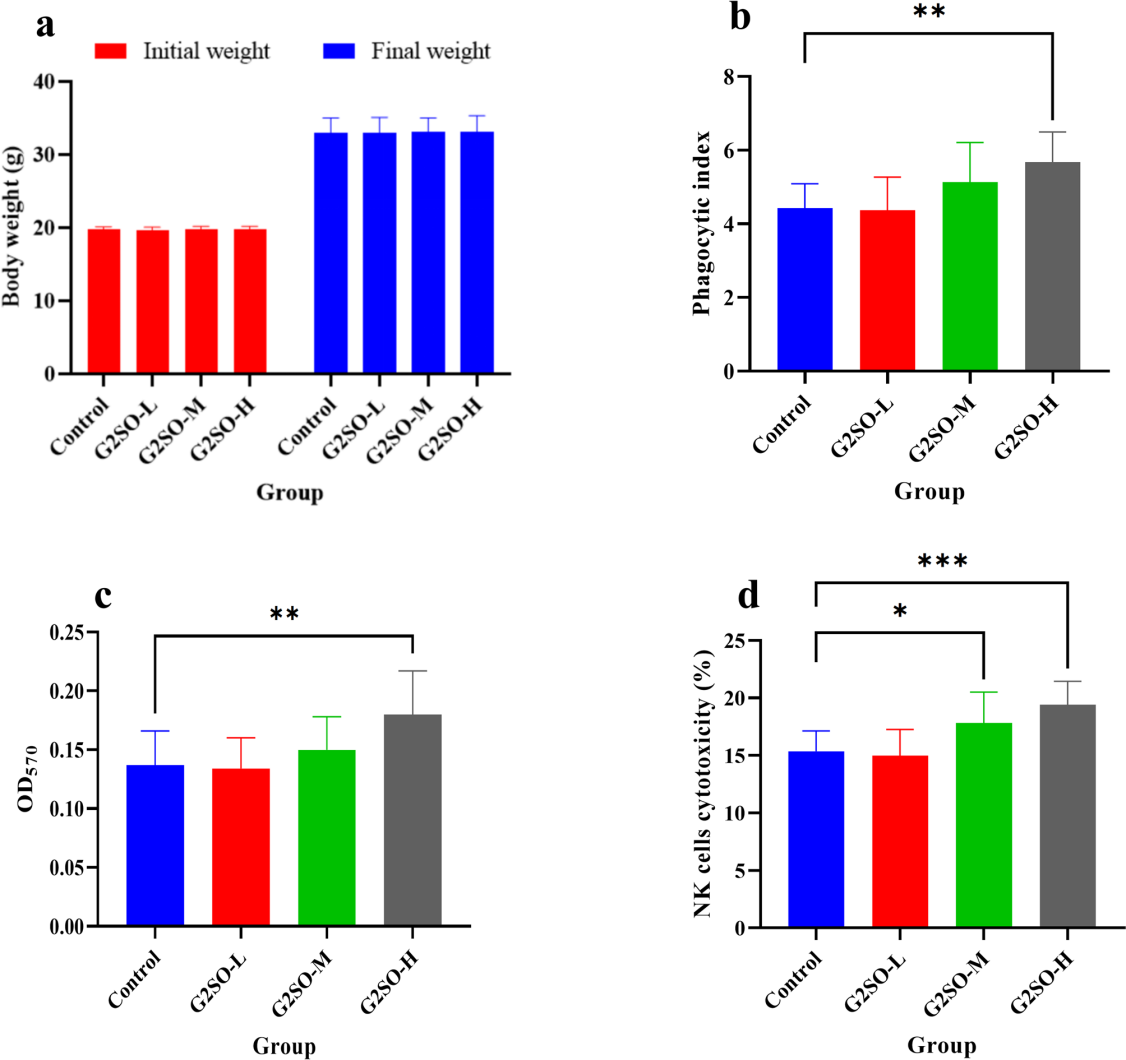
Effect of G2SO on body weight of mice treated with G2SO for 30 days (**a**), phagocytic index of macrophages measured by carbon clearance (**b**), proliferation of splenic lymphocytes by ConA induction (**c**), and NK cell cytotoxicity against YAC-1 cells (**d**). Data are expressed as mean ± SD. *n* = 10. The doses of G2SO-L, G2SO-M, and G2SO-H were 0.167, 0.333, and 1.0 g/kg bw/d oral gavage, respectively. These treatments were compared to the control. *P < 0.05, **P < 0.01, ***P < 0.001.

To confirm the effect of G2SO on body changes in mice, the body weight was recorded from the beginning until the end of the in vivo immune-enhancing experiment. In general, mice body weight increased from day 1 to day 30. Compared to the control, no significant changes (*P >* 0.05) were found on the body weight after 30 days of G2SO treatment (Fig. 3a). Immune organ indices are shown in Supplementary Fig. S1.

#### Effect of G2SO on carbon clearance

To investigate the influence of G2SO on macrophage activation, a carbon clearance assay was carried out. The results revealed that G2SO treatment, particularly at the high dose G2SO-H (5.68 ± 0.82), significantly (*P* ≤ 0.01) increased the phagocytic index compared to the control group (4.43 ± 0.66) (Fig. 3b). This indicates that G2SO enhanced the ability of macrophages to clear colloidal carbon (Indian ink) from the bloodstream.

#### Effect of G2SO on Splenic lymphocytes in mice

The proliferation of splenic lymphocytes was determined using ConA to stimulate T lymphocytes. After co-culture of ConA with splenic lymphocytes for 72 h, the proliferation of T lymphocytes to mitogen was detected as an important parameter for evaluating lymphocyte responsiveness. Compared with the control group (0.137 ± 0.029 OD_570_), T lymphocytes increased in the G2SO-treated groups, especially in the G2SO-H group (0.180 ± 0.037 OD_570_) were a significant difference (*P* ≤ 0.01) was observed (Fig. 3c).

#### Effect of G2SO on NK cell activity

The NK cell activity was determined to investigate the effect of G2SO on the innate immune function in mice. NK cells can identify the target cells ligands (e.g. YAC-1) and kill them by activating and inhibiting the signal integration of receptors^25^. NK cell activity in the G2SO treatment groups, specifically the G2SO-M (17.83 ± 2.69%) and G2SO-H (19.40 ± 2.05%) groups, was significantly increased (*P* ≤ 0.05) compared to that in the control group (15.36 ± 1.77%) (Fig. 3d).

#### Effect of G2SO on serum hemolysin level

**Fig. 4:**
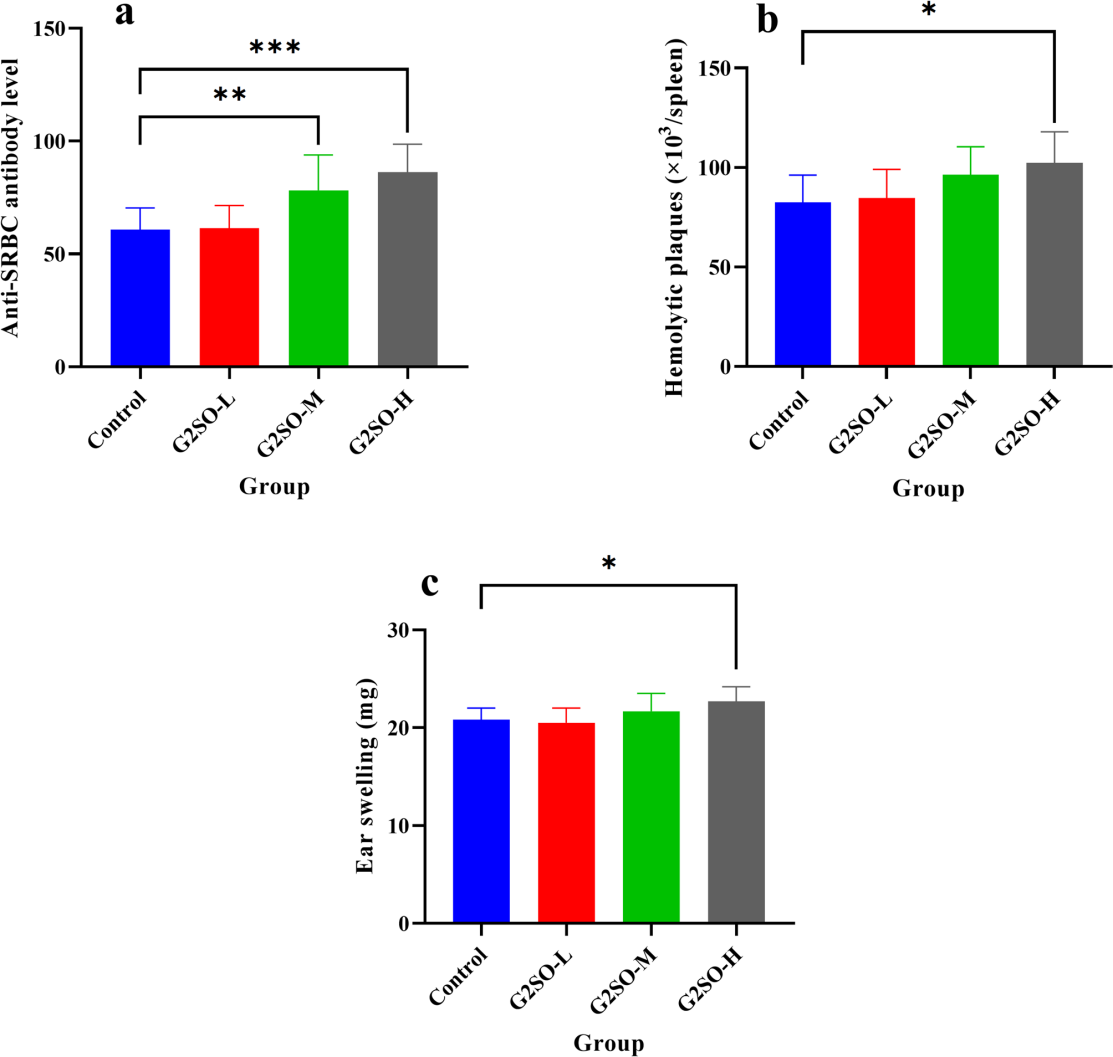
Effects of G2SO on humoral and cell-mediated immune responses in mice. Effect of G2SO on serum hemolysin level following SRBC injection in mice (**a**) serum hemolysin after SRBC immunization, measuring anti-SRBC antibodies in the mouse serum; (**b**) number of hemolysin-producing splenic cells measured by the modified Jerne’s slide method; (**c**) delayed type hypersensitivity (DTH) measured by DNFB induction in mice. Data are expressed as mean ± SD. *n* = 10. The doses of G2SO-L, G2SO-M, and G2SO-H were 0.167, 0.333, and 1.0 g/kg bw/d oral gavage, respectively. These treatments were compared to the control. *P < 0.05, **P < 0.01, ***P < 0.001.

To determine the effect of G2SO on the humoral immune response in mice, the changes in serum hemolysin levels were measured following SRBC injection. Hemolysin, an antibody produced by B lymphocytes, serves as a marker of humoral immunity. After a 30-day oral administration of G2SO, serum hemolysin levels were significantly increased (*P* ≤ 0.01) in the G2SO-M (78.2 ± 15.7) and G2SO-H (86.2 ± 12.5) treatment groups compared to that in the control group (60.8 ± 9.6) (Fig. 4a).

#### Effect of G2SO on hemolysin-producing cell response

To identify and count individual antibody-forming cells in the splenocytes of mice following treatment with G2SO, the modified Jerne’s slide method was carried out. This method allowed for the detection of hemolytic plaques, which are indicative of IgM-secreting antibody-forming cells. After a 30-day treatment with G2SO-M (96.42 ± 14.07 × 10^3^/spleen) and G2SO-L (102.30 ± 15.69 × 10^3^/spleen), the number of hemolytic plaques significantly increased (*P* ≤ 0.05) compared to the control group (82.43 ± 13.79 × 10^3^/spleen) (Fig. 4b).

#### Effect of G2SO on DTH

The degree of ear swelling, as measured by DTH, reflects the magnitude of the cellular immune response. To assess this, DNFB-induced ear edema was evaluated by applying a DNFB solution to the right ear of mice. Mice treated with G2SO-H (22.7 ± 1.5 mg) exhibited significantly greater (*P* ≤ 0.05) ear swelling compared to the control group (20.8 ± 1.2 mg), indicating a measurable enhancement of the cellular immune response (Fig. 4c).

### Exploratory in vivo evaluation of immune-enhancing effects in immunosuppressed mice

#### Effect of G2SO on serum cytokines

**Fig. 5:**
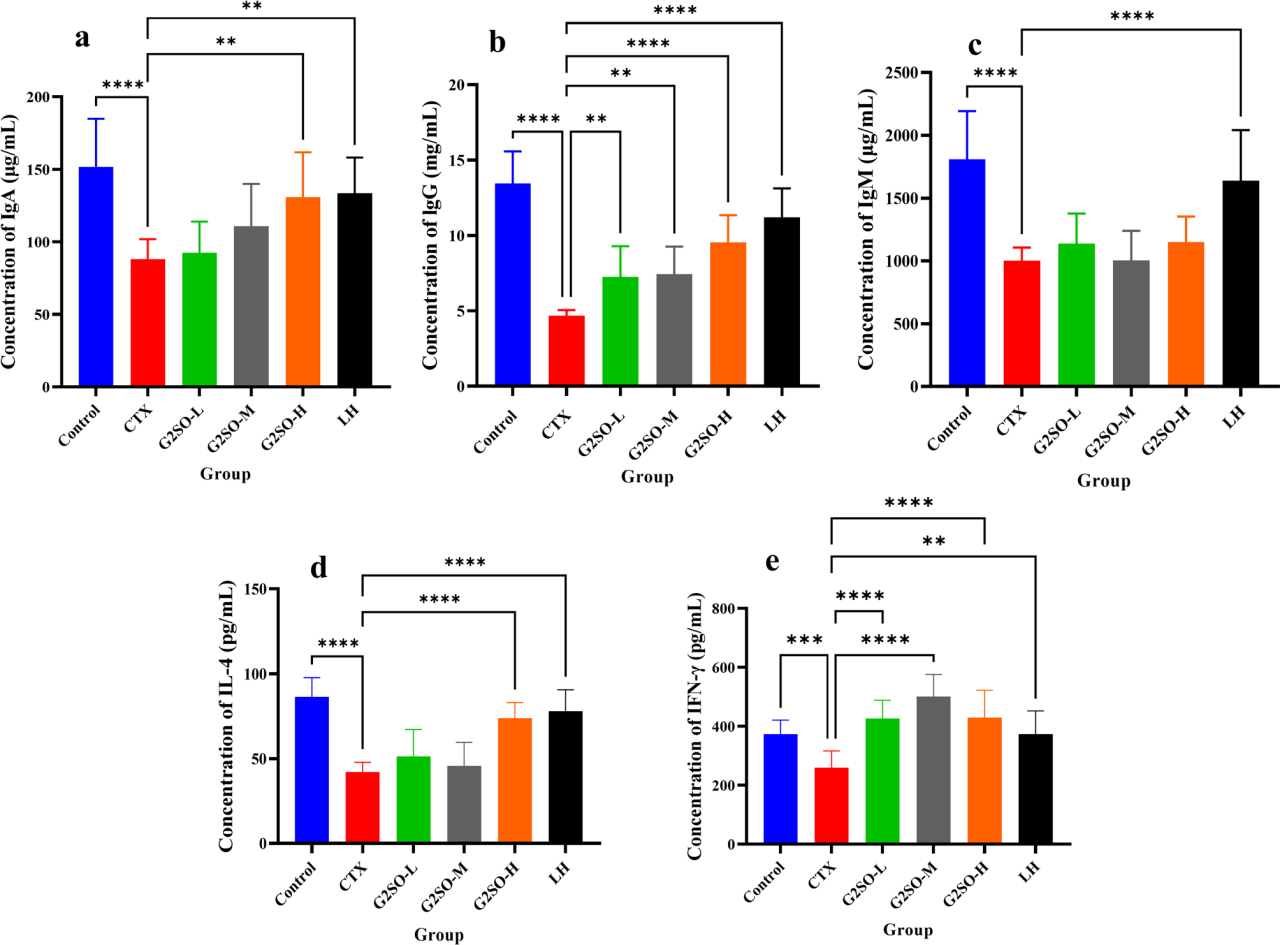
Effects of G2SO on serum immunoglobulins and cytokines in CTX-treated mice. (**a-c**) serum concentrations of immunoglobulins IgA (**a**), IgG (**b**), and IgM (**c**); (**d-e**) serum levels of cytokines IL-4 (d) and IFN-γ (**e**). Data are expressed as mean ± SD. *n* = 10. CTX, model group; LH, positive control (10 mg/kg bw/d oral gavage); the doses of G2SO-L, G2SO-M, and G2SO-H were 0.167, 0.333, and 1.0 g/kg bw/d oral gavage, respectively. All groups except control received intraperitoneal injection of CTX at 80 mg/kg bw/d from the 27th to 30th day. **P < 0.01, ***P < 0.001, ****P < 0.0001.

To determine the effect of G2SO on humoral immunity, the levels of IgA, IgG, and IgM, and the concentrations of IL-4 and IFN-γ in the serum of CTX-treated mice were detected (Fig. 5a-e). G2SO significantly (*P <* 0.001) increased the levels of IgA (130.85 ± 30.83 µg/mL), IgG (9.54 ± 1.80 µg/mL), IL-4 (73.61 ± 9.36 µg/mL), and IFN-γ (429.74 ± 92.98 µg/mL) in CTX-treated mice. The levels of IgA (87.97 ± 13.87 µg/mL), IgG (4.68 ± 0.37 µg/mL), IgM (1001.68 ± 103.99 µg/mL), IL-4 (42.16 ± 5.65 µg/mL), and IFN-γ (259.94 ± 56.67 µg/mL) in the CTX group (model group) were significantly (*P <* 0.001) lower than those in the control group.

#### Effect of G2SO on H&E examination of spleen and thymus in CTX-treated mice

**Fig. 6:**
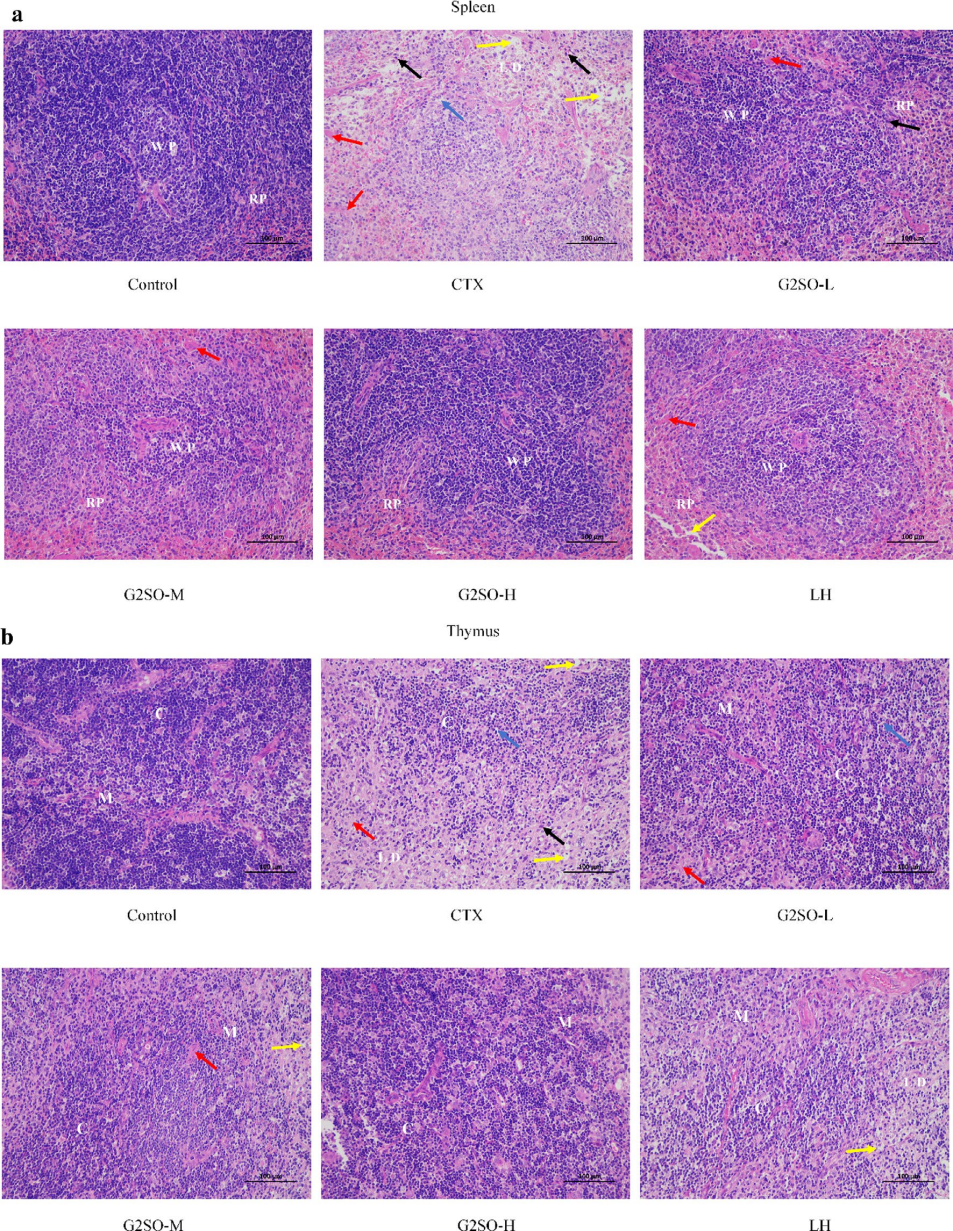

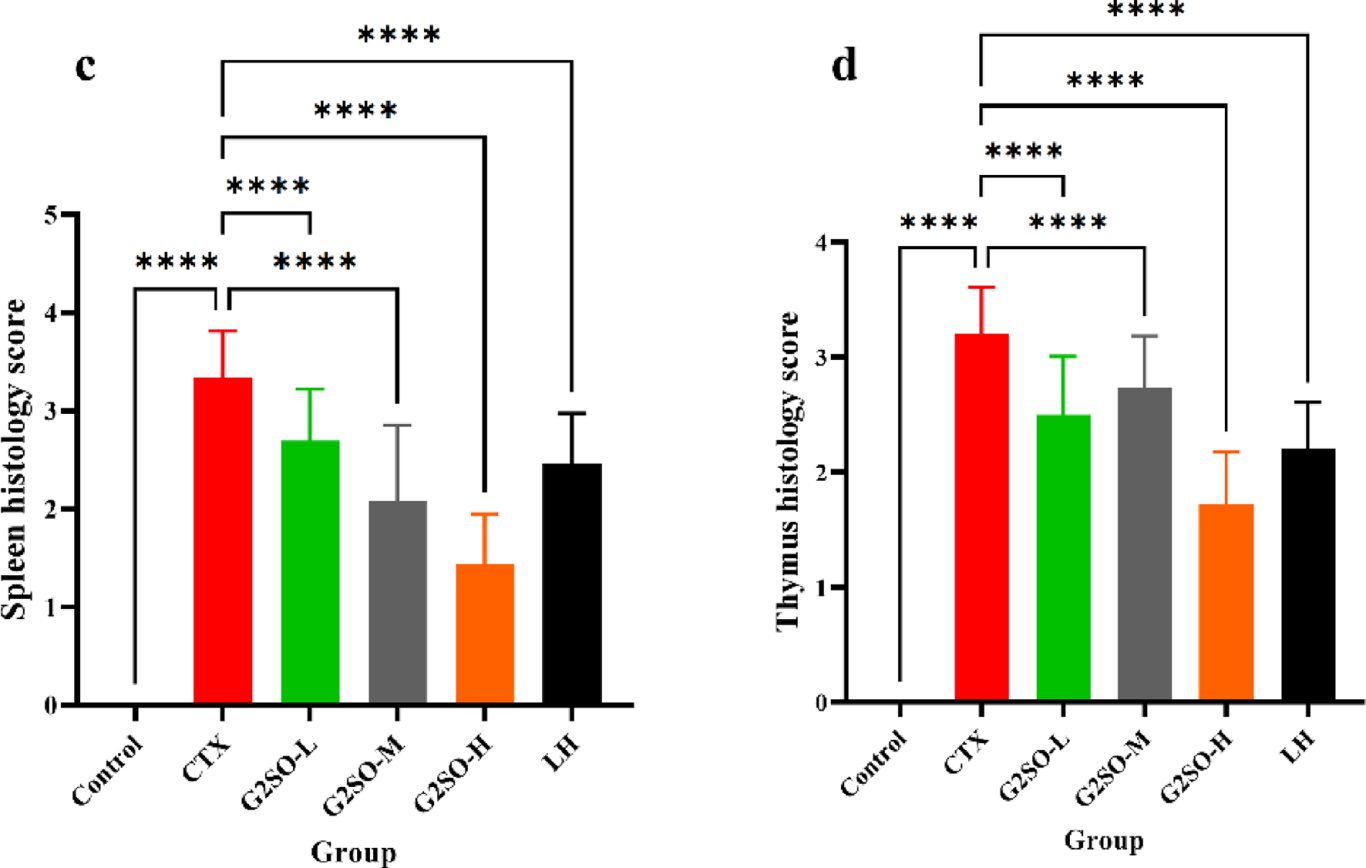
Representative images for H&E staining of spleen (**a**) and thymus (**b**) in CTX-treated immunosuppressed mice. Scale bar of 100 μm and magnification of 200×. Histological score of spleen (**c**) and thymus (**d**) for each group. CTX, model group; LH, positive control (10 mg/kg bw/d oral gavage); the doses of G2SO-L, G2SO-M, and G2SO-H were 0.167, 0.333, and 1.0 g/kg bw/d oral gavage, respectively. All groups except control received intraperitoneal injection of CTX at 80 mg/kg bw/d from the 27th to 30th day. WP: white pulp; RP: red pulp; LD: lymphocyte depletion; C: cortex; M: medulla; yellow arrow: vacuolization; blue arrow: swelling; red arrow: congestion; black arrow: inflammatory cell infiltration. ****P < 0.0001.

H&E staining revealed that the spleen in control group exhibited well-defined tissue structure, with regularly arranged lymphocytes and clearly demarcated red and white pulp (Fig. 6a). In contrast, the CTX group displayed a blurred white pulp with reduced area, sparse and diminished lymphocytes, and an overall disorganized cellular arrangement characterized by indistinct margins, pronounced intercellular space dilatation, and an unclear distinction between red and white pulp. Notably, spleen architecture was better preserved in the G2SO and LH groups, with clearer red-white pulp boundaries and more numerous, round lymphocytes.

In comparison to the control group, the model group exhibited marked thymic atrophy, cortical thinning, medullary enlargement, and irregularly shaped lymphocytes in the thymus (Fig. 6b). In contrast, mice in the G2SO-L, G2SO-M, G2SO-H, and LH groups showed improved thymic structure, with well-preserved lobules and a clear boundary between the cortex and medulla. Additionally, lymphocyte numbers were increased, and the cells appeared more uniformly round. Histological scoring revealed significantly (*P* < 0.05) greater spleen and thymus injury in the CTX group than in the control group, whereas G2SO treatment significantly (*P* < 0.05) attenuated these lesions compared with CTX group (Fig. 6c-d).

### In vitro evaluation using RAW264.7 cells

#### Effect of G2SO on RAW264.7 cell viability

**Fig. 7:**
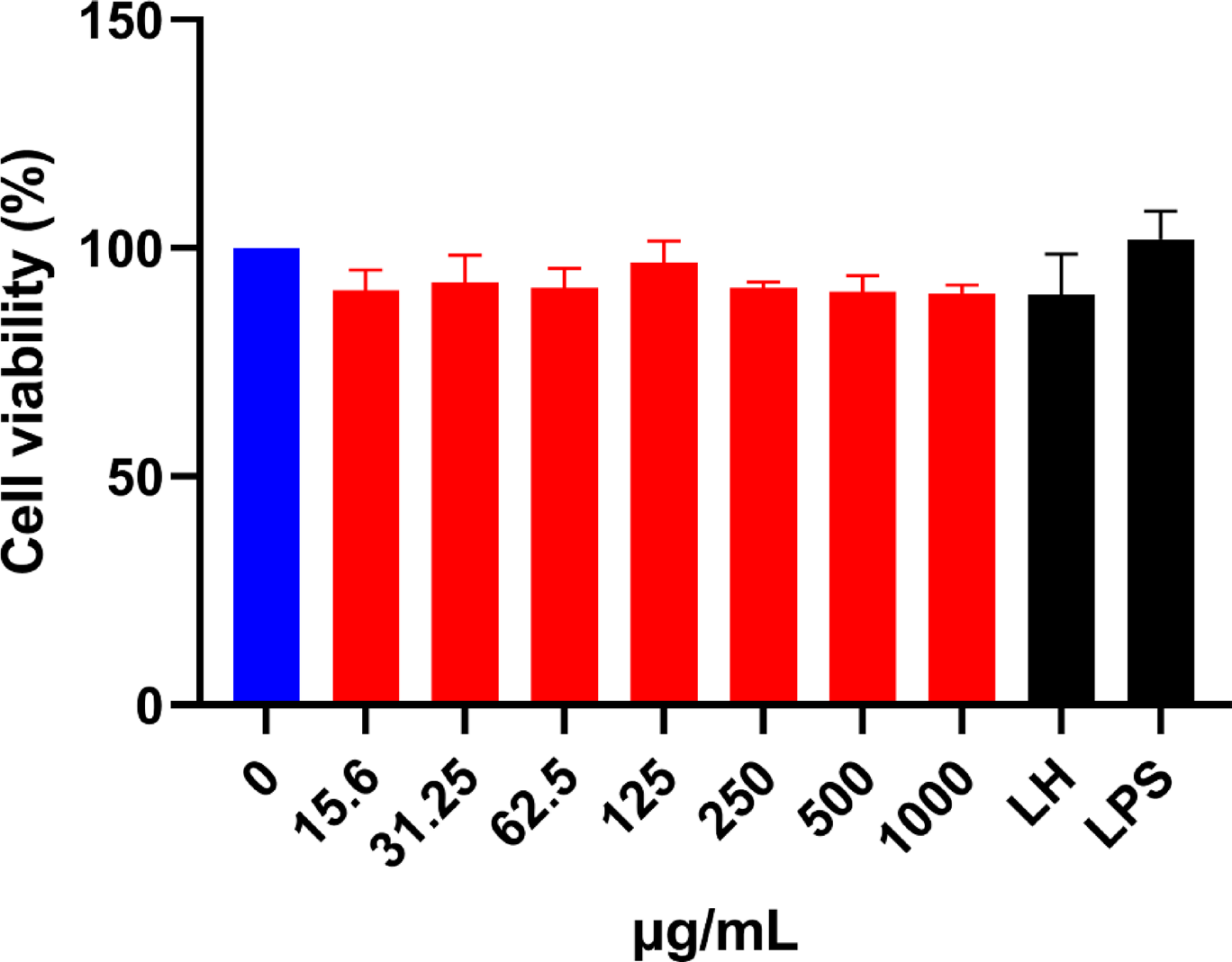
Effect of G2SO on the viability of RAW264.7 cells. RAW264.7 cells were treated with G2SO at the indicated concentrations, LH (10 nmol/mL), or with LPS (1 µg/mL) for 24 h. Cell viability was assessed by MTT assay. Data are expressed as mean ± SD. *n* = 3. No significant differences (*P* > 0.05) were found.

**Fig. 8:**
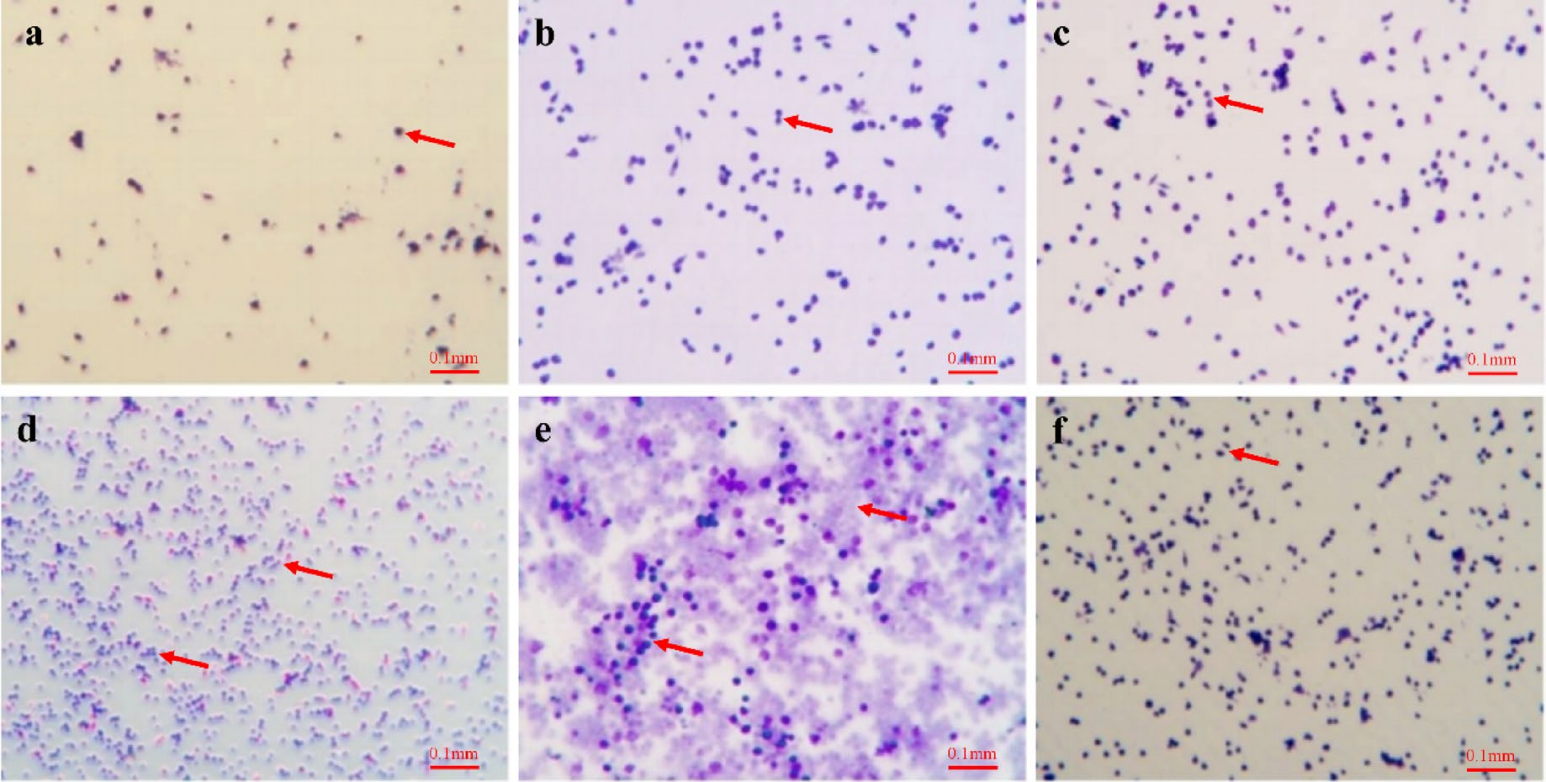
Effects of G2SO on the adhesion function of RAW264.7 cells. Cells were seeded in 24-well plates for 1 h. a: control. b-e: 125, 250, 500, 1000 µg/mL G2SO, respectively. f: 1 µg/mL LPS. Cells were observed under a microscope under a magnification of 40×. Red arrows show adhered cells.

The cytotoxic effects of G2SO on RAW264.7 cells were evaluated using the MTT assay. No significant differences were observed in cells treated with G2SO compared to the control (Fig. 7). The results suggest that G2SO did not adversely affect the health or proliferation of the RAW264.7 cells within the concentrations tested. RAW264.7 cells displayed cell viability between 90.11 ± 1.86% and 96.77 ± 4.74%. In mice, blood volume (volume of distribution) is ~ 72–80 mL/kg; assuming oral bioavailability (F) of 5–10% for a poorly water-soluble lipidic mixture (G2SO), the theoretical maximum concentration is: Theoretical concentration = (dose×F) / volume of distribution = (1000 mg/kg×0.05–0.10) / (72–80 mL/kg) ≈ 0.6–1.4 mg/mL. Thus, 1 mg/mL was used as a maximum concentration, with lower concentrations spanning sub-physiologic exposures. These in vitro assays are mechanistic screens rather than PK-matched simulations, and extensive protein binding and tissue distribution mean that in vitro nominal concentrations are not directly comparable to free plasma levels in vivo (Fig. 8).

#### Effect of G2SO on cytokine production in RAW264.7 cells

**Fig. 9:**
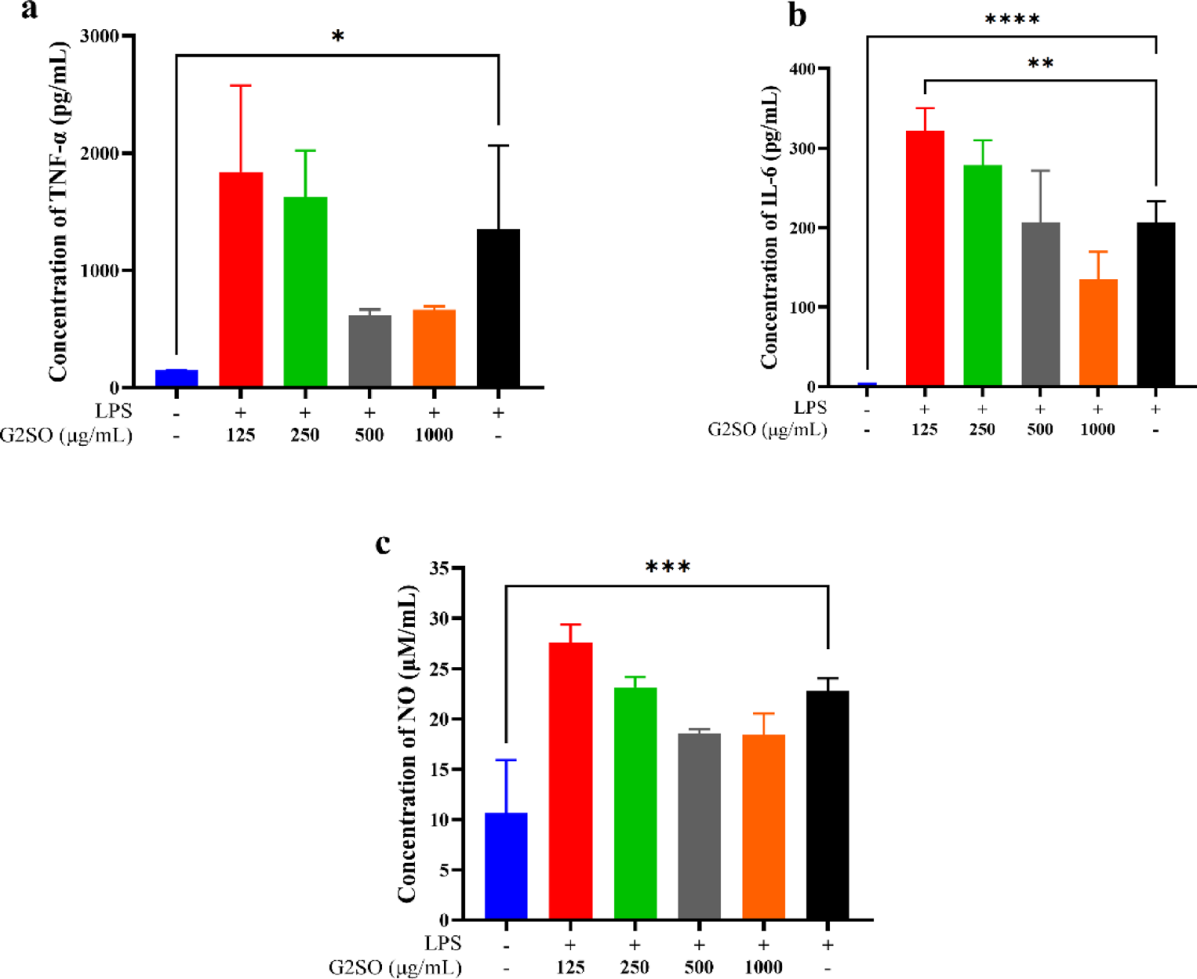
Effect of G2SO on pro-inflammatory mediator release in LPS-stimulated RAW264.7 macrophages. TNF-α (**a**), IL-6 (**b**), and NO (**c**). RAW264.7 cells were simultaneously treated with LPS and G2SO at 125, 250, 500, or 1000 µg/mL for 24 h. All treatments except the control were treated with 1 µg/mL LPS. Data are expressed as mean ± SD. *n* = 3. *P < 0.05, **P < 0.01, ***P < 0.001, ****P < 0.0001.

To evaluate the potential immunomodulatory effect of G2SO on RAW264.7 macrophages, the levels of TNF-α, IL-6, and NO were measured. Treatment with G2SO attenuated LPS-stimulated production of TNF-α, IL-6, and NO (Fig. 9a-c). IL-6 secretion was significantly reduced at the lower G2SO dose (*P* ≤ 0.05 vs. LPS), while changes in TNF-α and NO and at the higher dose did not reach statistical significance, despite showing downward trends. Thus, no clear dose-response was detected under these conditions.

#### Effect of G2SO on adhesion function of RAW264.7 cells

The effect of G2SO on the immune function of RAW264.7 cells was assessed by evaluating the adhesion capacity of these cells following exposure to G2SO. Adhesion of macrophage-like RAW264.7 cells is a crucial function for initiating immune responses, as it enables the cells to interact with pathogens, release cytokines, and initiate phagocytosis. G2SO exposure produced a concentration-related increase in RAW264.7 adhesion under the assay conditions; therefore we describe the change descriptively without inferring function (Fig. 8).

## Discussion

Natural products have attracted considerable interest in cancer control and immunomodulation^26^. TCM, long practiced in China and other parts of Asia, may offer potential candidates for supporting immune function and cancer prevention^13,27^. *Ganoderma*, one of the most extensively studied TCM and food homologous fungi, has been shown in numerous studies to contain natural compounds that play a crucial role in treating a variety of human diseases. In this study, the contents of ergosterol and total triterpenes in G2SO were similar to those in *G. lucidum* spore oil, which was reported to prevent breast cancer lung metastasis and to modulate immune activity in mice by promoting splenocyte proliferation and supporting NK cell activity^8^. Moreover, G2SO contains polysaccharides which are from the fruiting body extracts of *G. lucidum* and *G. sinense*. Generally, these compounds are believed to be responsible for the antitumor and immunomodulatory effects of *Ganoderma*. In this study, G2SO reduced the growth of 4T1 transplant tumors in mice under the conditions tested, suggesting in vivo antitumor effects. The combination of spore oil and fruiting body extract is thought to offer a synergistic approach in cancer control and immune system enhancement. The bioactive compounds in spore oil (notably triterpenes and ergosterol) together with β-glucan-rich polysaccharides in the fruiting body extract likely act through defined cellular processes. First, *Ganoderma* polysaccharides engage macrophage pattern-recognition receptors (notably TLR4), activating NF-κB/MAPK signaling and increasing NO and IL-6, TNF-α cytokines, which is consistent with our dose-dependent cytokine/NO increases^28,29^. Second, this innate activation promotes antitumor immunity by enhancing NK-cell cytotoxicity and Th1 responses (IL-12/IFN-γ)^30,31^. Third, *Ganoderma* triterpenoids and spore oil trigger mitochondrial apoptosis with caspase-9/3 activation and cell-cycle arrest in tumor cells, providing a mechanistic basis for the reduced tumor burden observed^32,33^. Together, these pathways provide a plausible mechanistic context for the observed antitumor activity and associated immunomodulatory effects of G2SO.

Unlike previous studies, G2SO combines a triterpene-rich lipid phase (spore oil) with polysaccharide-rich aqueous extracts from *G. lucidum* and *G. sinense*; this likely dilutes per-dose triterpenoid exposure relative to solvent-enriched fractions that drive direct cytotoxicity, whereas polysaccharides primarily act as immunomodulators^34^. In the present study, (i) both innate and adaptive immune functions were profiled in a single study (NK activity, macrophage phagocytosis, splenocyte proliferation to mitogens, cytokines, and T-cell subsets); (ii) two physiological contexts were evaluated—normal and CTX-immunosuppressed mice—together with therapeutic efficacy in 4T1 tumor-bearing mice; and (iii) tolerability at the tested doses was documented. To our knowledge, this integrated design and this specific formulation have not been reported. Prior studies vary in solvent, fungal part (spores vs. fruiting bodies), and species/strains, which alter lanostane-type triterpenes and β-glucan yields^35^. Collectively, these factors align with our observation that G2SO’s primary antitumor action is immunological, consistent with literature showing that *Ganoderma* polysaccharides enhance NK/T-cell function and macrophage activity^34^.

Mechanistically, the triterpenes and ergosterol in *G. lucidum* spore oil have been shown to trigger mitochondrial-mediated apoptosis in cancer cells through upregulation of Bax, and activation of caspase-3 and caspase-9^32,36^. Ergosterol further suppresses the expression of pro-inflammatory mediators through several signaling pathways^37^. Polysaccharides from the fruiting bodies are notable immunomodulators. Polysaccharides boost immune function by acting on key immune cells, macrophages, B and T lymphocytes, NK cells, and dendritic cells. In particular, *G. lucidum* polysaccharides promote T- and B-cell growth by binding to Toll-like receptor 4 (TLR4). When *G. lucidum* polysaccharides engage TLR4 or TLR2 on these cells, they activate a signaling pathway that goes through p38 MAP kinase and downstream induction of immunoglobulin production and cytokines^38,39^. In parallel, β-glucan-rich polysaccharides activate the C-type lectin receptor Dectin-1, which signals via Syk-CARD9 and converges on NF-κB; this pathway can cooperate with TLRs to amplify cytokine responses^40^. Therefore, resulting in macrophage and dendritic-cell activation and subsequent modulation of adaptive immunity. *G. lucidum* polysaccharides can inhibit tumor cell proliferation, induce oxidative stress, and modulate inflammatory mediators and reactive oxygen species in cancer models^41^. In G2SO, the combination of triterpenes and ergosterol (from spore oil) with polysaccharides (from fruiting body extracts) likely yields synergistic antitumor actions: the lipophilic fraction drives direct cytotoxicity and apoptosis, while polysaccharides exert anti‐proliferative and anti‐angiogenic effects, as well as immunostimulation. Taken together, these reported activities provide a plausible framework by which G2SO could contribute to tumor suppression. Further mechanistic studies in tumor-bearing models are required to establish causality between the observed immune changes and tumor reduction.

Moreover, circulating neutrophils fluctuate with release from bone marrow, intravascular margination/demargination, and tissue homing. Accordingly, a reduction in neutrophils associated with G2SO may reflect altered trafficking or a lower inflammatory drive rather than impaired granulopoiesis^42^.

The immune system is a complex and precise network composed of immune organs, cells, and active substances^43^. It consists of both innate and adaptive immunity, which together provide the body’s primary defense against infections^21^. The thymus and spleen are the major immune organs in the human body and indicate overall immune function^24^. The thymus plays a critical role in T-cell maturation, while the spleen is essential for filtering blood and mounting immune responses^44,45^.

Innate immunity comprises a range of defense mechanisms that respond quickly to invading pathogens and play a pivotal role in initiating and modulating specific immunity. Immune cells such as NK cells and macrophages regulate the innate immune response^31^. Peritoneal macrophages are capable of phagocytizing viruses and bacteria, activating other immune cells, and stimulating the release of various immune factors and cytokines, such as NO, IL-6, and TNF-α^43,46^. NK cells, primarily residing in the spleen, are key components of innate immunity and possess the ability to kill abnormal cells, pathogen-infected cells, and tumor cells^21^. As such, both macrophages and NK cells are crucial in the body’s antimicrobial defense and pathogen response^47^. Although no significant differences were observed in the peritoneal macrophage phagocytosis and phagocytic index, there was an increasing trend associated with the higher concentrations of G2SO. However, G2SO significantly increased the macrophage phagocytic index as shown in the carbon clearance assay, which evaluates the effect of G2SO on macrophage-mediated clearance of carbon from the blood and assesses the function of the reticuloendothelial system, a network of phagocytic cells involved in immune response^46^. Enhanced macrophage-mediated clearance is a key indicator of improved immune response, as it reflects the activation of the reticuloendothelial system and the ability to eliminate foreign particles and pathogens from the body. The increase in carbon clearance suggests an augmentation of macrophage activity and overall immune function following 30 days of G2SO administration.

LPS, an endotoxin from Gram-negative bacteria, is commonly used in experimental models of systemic bacterial infection and inflammation, triggering the release of TNF-α, IL-6, and NO^48,49^. The secretion of NO serves as an indicator of RAW264.7 macrophage activation^24^. TNF-α, secreted by activated macrophages, promotes the production of other inflammatory mediators, while IL-6 stimulates adaptive immune responses by promoting T and B cell differentiation^50^. While the release of inflammatory mediators is essential for combating infections, excessive amounts can lead to collateral damage to normal cells^48^. In this study, G2SO increased TNF-α, IL-6, and NO at low concentrations, followed by attenuation at higher doses. Such a biphasic (hormetic) response is characteristic of macrophage regulation, where low-intensity stimuli enhance innate activation while stronger inputs engage counter-regulatory pathways^51^. Taken together, these in vitro results suggest that G2SO may modulate macrophage activation in a stimulus-dependent manner under the tested conditions.

To assess the effect of G2SO on NK cell activity, YAC-1 cells were used as target cells and splenocytes as effector cells. We found significant differences between the control and G2SO-fed groups, particularly at medium and high doses, which corresponded to enhanced NK cell cytotoxic activity against the target cells. This increased cytotoxic activity against YAC-1 cells suggests that G2SO may be associated with enhanced NK-like cytotoxicity ex vivo, which could contribute to antitumor activity. However, a causal immune-mediated mechanism was not directly tested in this study.

Adaptive (specific) immunity provides a more efficient defense against non-self antigens than innate immunity. It engages both cellular and humoral mechanisms, mediated by T lymphocytes and B lymphocytes, respectively. These processes are tightly regulated through interactions with antigen-presenting cells, allowing for the targeted elimination of pathogens^52^. In this study, immunization with G2SO led to the secretion of hemolysin by B lymphocytes, with serum hemolysin levels used as an indicator of humoral immunity^21^. These results suggest that G2SO can effectively enhance humoral immune activity in mice by stimulating B cell-mediated antibody production. Moreover, G2SO may enhance the immune response by stimulating IgM-secreting cells in mice, thereby promoting humoral immunity^53^. B-cell-related assay was included to determine general adaptive immunity after G2SO administration. Because we did not measure tumor-antigen–specific antibodies, tumor-infiltrating B cells, or perform B-cell depletion, these data should be interpreted as immune enhancement markers rather than evidence that B cells are required for the antitumor effect.

When innate immunity is insufficient to block pathogen invasion, adaptive immunity generates T lymphocytes to resolve the infection^52^. Although the number of splenocytes increased following exposure to the mitogen ConA, the increases observed here may partly arise from G2SO-related changes in lymphocyte composition, because ConA induces non-specific T lymphocytes proliferation. Therefore, there is a possibility that G2SO may increase T lymphocyte number when the body is exposed to foreign invaders, indicating that G2SO-H was able to possibly increase the proliferative capacity of T lymphocytes and potentially enhance the immune system. Moreover, the enhanced ear swelling after G2SO treatment further confirms enhancement of T-cell-mediated immunity, which is a key feature of the cellular immune function.

In CTX-treated mice, CTX induces profound immunosuppression, marked by significant reductions in serum immunoglobulins (IgA, IgG, IgM) and cytokines (IL-4, IFN-γ)^43^. *Ganoderma* polysaccharides have been shown to counteract these effects by promoting the formation of IgA-secreting cells and upregulating Toll-like (TLR-2/4), thereby restoring IgA, IgG, and IgM levels in CTX models^54^. Our data demonstrate significant restoration of IgA, IgG, IL-4, and IFN-γ levels with G2SO treatment thus aligning with these previous findings. Together, these results indicate that G2SO may exert a protective effect against CTX-induced immunosuppression by reversing declines in both immunoglobulins and critical immune mediators. While CTX models provide a clinically relevant immunosuppressed context, their immune effects are complex and dose-dependent; therefore, findings should be integrated with future mechanistic studies in tumor-bearing mice.

Histopathological examination of the spleen and thymus, together with comprehensive blood cell counts and serum biochemical parameters, showed that no overt G2SO-related toxicity was observed in mice within the limits of the study design. No obvious morphological alterations were detected in these immune organs, indicating that G2SO did not compromise immune integrity in this short-term study. In vitro MTT assay in RAW264.7 cells showed that G2SO did not cause apparent cytotoxicity at the tested concentrations. Moreover, G2SO enhanced RAW264.7 cell adhesion, which may reflect macrophage activation and a potentially improved responsiveness to immune challenges.

The observed antitumor and immunomodulatory effects of G2SO are likely due to its bioactive components, such as polysaccharides and triterpenes, which interact with immune cell receptors and modulate signaling pathways^31^. Immunomodulatory effects in normal and immuno-compromised mice, together with the absence of overt toxicity within the limits of the study design, supports the potential of G2SO as a functional health product that can activate immune responses without compromising cell integrity. These findings are consistent with the traditional use of *Ganoderma* for promoting health and highlights its potential for modern applications in functional foods and nutraceuticals.

## Conclusions

In conclusion, G2SO exhibited measurable antitumor activity in the 4T1 breast cancer model and was associated with modest changes in several innate and adaptive immune parameters in mice under the tested conditions. These findings support G2SO as a candidate immunomodulatory and cancer control formulation warranting further mechanistic and translational evaluation. However, our study has limitations. Modest group sizes and biological variability may have contributed to dispersion in some endpoints. In addition, G2SO is a complex mixture that was partially characterized chemically; further work should isolate and quantify active constituents, elucidate mechanisms of action, and evaluate clinical relevance. The antitumor study was performed in female BALB/c mice, whereas immunological experiments used male ICR mice, so sex- and strain-specific responses were not controlled within a single framework. Although immune assays indicate functional enhancement, mechanistic depth was limited because comprehensive immune phenotyping and signaling-pathway analyses were not performed. Immune enhancement in immunosuppressed mice was exploratory. Because ConA induces non-specific T lymphocytes proliferation, the observed increases may partly reflect changes in lymphocyte composition; multiparameter flow cytometry was not performed after G2SO administration, so these results should be interpreted with caution. Future studies should phenotype T-, B-, NK- and myeloid subsets and assess activation/proliferation markers to distinguish compositional from intrinsic effects. While we observed antitumor activity accompanied by systemic immunomodulatory changes, causal mechanisms, particularly within the tumor microenvironment, remain to be established and should be addressed in tumor-bearing models via profiling of tumor-infiltrating immune subsets and functional perturbation experiments. Finally, because this study was not designed as a formal head-to-head comparison with *G. lucidum* spore oil, firm conclusions about relative efficacy cannot be drawn; randomized, dose-matched studies using harmonized source materials and analytical standardization will be required to determine whether G2SO provides superior advantages over spore oil alone.

## Supplementary Information

Below is the link to the electronic supplementary material.

Supplementary Material 1

## Data Availability

The datasets used and/or analyzed during the current study are available from the corresponding author on reasonable request.
