# Supplementary material for: Ganoderma lucidum spore oil with Ganoderma lucidum and Ganoderma sinense extracts (G2SO) shows antitumor and Immunomodulatory effects in mice
Source: Sci Rep. 2026 Jan 5;16:4751. doi: 10.1038/s41598-025-34803-9 (PMC12873310; doi:10.1038/s41598-025-34803-9)
Supplement: Supplementary file 1 — Supplementary Material 1 [file 41598_2025_34803_MOESM1_ESM.docx]

**Supplementary Material**

**Supplementary Table S1.** Batch-level contents of main bioactive compounds in G2SO (32 batches).

| Batch number | Crude polysaccharides (g/100 g) | Total triterpenes (g/100 g) | Ganoderic acid A (mg/100 g) | Ergosterol (mg/100 g) |
| --- | --- | --- | --- | --- |
| 2402008 | 7.8 | 34.1 | 76 | 216 |
| 2403010 | 7.6 | 33 | 77.5 | 236 |
| 2404006 | 7.9 | 33.9 | 72.1 | 235 |
| 2405020 | 7.6 | 32.7 | 74.6 | 199 |
| 2405025 | 7.5 | 31.5 | 74.9 | 200 |
| 2406002 | 7.8 | 33.4 | 75.6 | 219 |
| 2406006 | 7.9 | 34.1 | 76 | 213 |
| 2406012 | 7.9 | 39.8 | 77.9 | 213 |
| 2407009 | 6.9 | 36.6 | 78.9 | 206 |
| 2407022 | 8.1 | 32.4 | 74.8 | 211 |
| 2409001 | 8.3 | 33.6 | 77.5 | 237 |
| 2409008 | 8.2 | 36.3 | 79.1 | 230 |
| 2409016 | 8.3 | 34.8 | 82.8 | 233 |
| 2410013 | 8.0 | 32.6 | 78.9 | 252 |
| 2411010 | 8.2 | 35.2 | 72 | 225 |
| 2411028 | 8 | 34.2 | 84.5 | 225 |
| 2412007 | 8.1 | 35.9 | 86.2 | 221 |
| 2412021 | 8.6 | 37 | 88 | 220 |
| 2501001 | 8.1 | 35.7 | 94.3 | 221 |
| 2501016 | 8.2 | 34.4 | 93.4 | 218 |
| 2502018 | 8.1 | 34.4 | 88.5 | 227 |
| 2503002 | 7.5 | 34.7 | 95.4 | 225 |
| 2503014 | 7.4 | 32.0 | 93.5 | 221 |
| 2503018 | 7.5 | 33.4 | 93.8 | 222 |
| 2505003 | 7.3 | 31.7 | 96.8 | 221 |
| 2505023 | 7.7 | 31.0 | 87.9 | 224 |
| 2506009 | 7.3 | 31.6 | 86.1 | 224 |
| 2506019 | 7.7 | 31.7 | 94.8 | 267 |
| 2507020 | 7.7 | 32.1 | 88.5 | 223 |
| 2509006 | 7.3 | 32.2 | 101 | 220 |
| 2510006 | 8.0 | 31.1 | 95 | 225 |
| 2511008 | 7.8 | 32.1 | 91.7 | 220 |

**b**

**a**

**Supplementary Fig. S1.** Effect of G2SO on spleen index (a) and thymus index (b) in mice. Data are expressed as mean ± SD. The doses of G2SO-L, G2SO-M, and G2SO-H were 0.167, 0.333, and 1.0 g/kg bw/d oral gavage, respectively. *n*=10. These treatments were compared to the control.


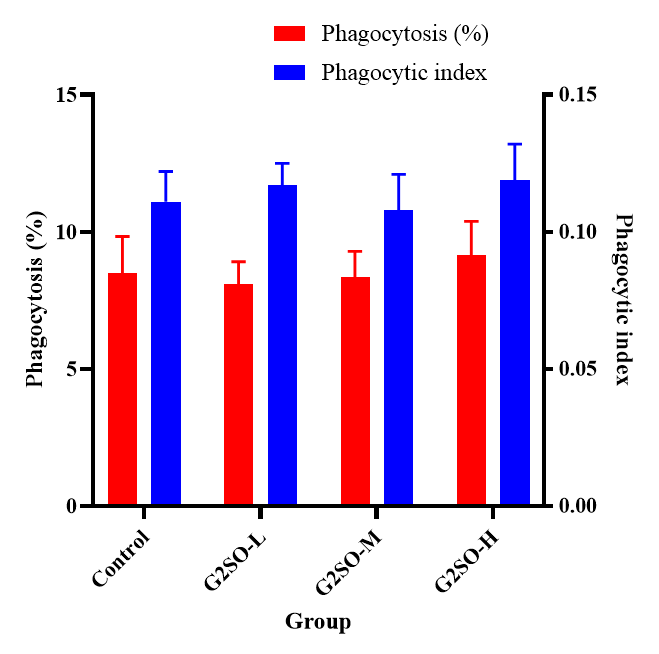


**Supplementary Fig. S2.** Effect of G2SO on phagocytosis (%) and phagocytic index of peritoneal macrophages. Data are expressed as mean ± SD. *n* = 10. The doses of G2SO-L, G2SO-M, and G2SO-H were 0.167, 0.333, and 1.0 g/kg bw/d oral gavage, respectively. These treatments were compared to the control.
